# Hydrocarbon Sorption in Flexible MOFs—Part I: Thermodynamic Analysis with the Dubinin-Based Universal Adsorption Theory (*D-UAT*)

**DOI:** 10.3390/nano12142415

**Published:** 2022-07-14

**Authors:** Hannes Preißler-Kurzhöfer, Marcus Lange, Andrei Kolesnikov, Jens Möllmer, Oliver Erhart, Merten Kobalz, Harald Krautscheid, Roger Gläser

**Affiliations:** 1 Institut für Nichtklassische Chemie e.V., Universität Leipzig, Permoserstraße 15, D-04318 Leipzig, Germany; che09bcz@studserv.uni-leipzig.de (H.P.-K.); lange@inc.uni-leipzig.de (M.L.); kolesnikov@inc.uni-leipzig.de (A.K.); moellmer@inc.uni-leipzig.de (J.M.); 2 Institut für Technische Chemie, Fakultät für Chemie und Mineralogie, Universität Leipzig, Linnéstraße 3, D-04103 Leipzig, Germany; 3 Institut für Anorganische Chemie, Fakultät für Chemie und Mineralogie, Universität Leipzig, Johannisallee 21, D-04103 Leipzig, Germany; oliver.erhart@uni-leipzig.de (O.E.); merten.kobalz@uni-leipzig.de (M.K.); krautscheid@rz.uni-leipzig.de (H.K.)

**Keywords:** metal–organic frameworks, thermodynamic analysis, flexible materials

## Abstract

The analysis of empirical sorption equilibrium datasets is still vital to gain insights into material–property relationships as computational methods remain in development, especially for complex materials such as flexible MOFs. Therefore, the Dubinin-based universal adsorption theory (*D-UAT*) was revisited and evaluated as a simple visualization, analysis, and prediction tool for sorption equilibrium data. Within the theory, gas properties are normalized into corresponding states using the critical temperatures of the respective sorptives. The study shows theoretically and experimentally that the *D-UAT* is able to condense differences of sorption data visualized in reduced Dubinin plots to just three governing parameters: (a) the accessible pore volume, (b) the reduced enthalpy of sorption, and (c) the framework’s reduced free energy differences (in case of flexible behavior). This makes the theory a fast visualization and analysis tool, the use as a prediction tool depends on rough assumptions, and thus is not recommended.

## 1. Introduction

Flexible metal–organic frameworks (MOFs) have the ability to change their structure, including the molecular conformation of the organic linkers upon external stimuli such as temperature [1], mechanical pressure [2], electric fields [3,4], but, most importantly, due to the adsorption of guest molecules [5,6,7]. This makes flexible MOFs an intriguing subclass of porous materials for applications like gas separation and storage [8,9,10,11], catalysis [12], or sensor design [13], as well as drug delivery [14]. Despite the already large number of MOF structures (Cambridge structural database lists above 60,000 MOF structures currently [15]), the modularity of the building blocks, inorganic linkers, and metal or cluster nodes makes a complete exploitation of all structural possibilities experimentally unfeasible. In order to enable a directed research of MOF materials suited for specific applications within adsorption technology, computational simulations are more and more deployed for the precise prediction of limiting pore widths, accessible pore volumes or complete sorption equilibria data. For rigid materials, approaches like grand-canonical Monte Carlo simulations already showed great prediction accuracy [16,17]. For flexible MOFs, computationally much more demanding calculations of free energy profiles for complex adsorptive–adsorbent systems are necessary. These calculations are able to predict the complex sorption isotherms and thus became a focal topic of research within the last years [18,19,20]. However, these simulations are usually based on ab initio methods and are computationally extremely expensive as of now [21].

Thus, the prediction of sorption isotherms was approached via the analysis of existing datasets by several groups. Yamazaki et al. utilized the well-known Dubinin approach to normalize various isotherms in a wide temperature range into working pair specific characteristic curves using the flexible MOF (Cu(dhbc)_2_(4,4′-bpy)) and various fluids such as Xe, Ar, CO_2_, and CH_4_ [22]. It was found that, although the characteristic curves for different gases deviated in their respective positions within the Dubinin plot, the overall shapes remained the same. Furthermore, a distinct relationship between the gate-opening pressures and the enthalpy of adsorption was drawn, however, without deeper analysis.

In reaction to that, Sircar et al. [23] combined Dubinin’s theory with the relatively new universal adsorption theory (*UAT*) by Quinn [24] for the same system studied by Yamazaki et al. Herein, the critical temperatures of the gases were used as scaling factors of the sorption potential. It was based on the assumption that a complete coincidence of all isotherms within a Dubinin plot would lead to a universal pattern for one specific adsorbent, from which every isotherm for any adsorptive could be derived. The complete coincidence was not observed, but a minor qualitative agreement was shown for gases in supercritical states within this work [23].

This raises the question to what the Dubinin-based universal adsorption theory, from hereon called *D-UAT*, is capable of, since experimental and theoretical proof of its applicability or boundaries thereof is still missing to the best of our knowledge, regardless of rigid or flexible materials. This may be due to the fact that most studies are predominantly concerned with chemically very different fluids such as CO_2_, N_2_, CH_4_, or noble gases, which in turn pose very different adsorbent–adsorptive interactions or even quantum effects, making a more general investigation rather challenging [25].

Thus, this study intends to achieve the following objectives:

1. Applying the *D-UAT* on an empirical sorption data set with several adsorbents, adsorptives, and temperatures, and evaluate the practical abilities of the theory for visualization, analysis, and prediction of sorption isotherms for both rigid and flexible materials
2. Revisit the *D-UAT* from a purely theoretical point of view and find mathematical proof of its applicability for visualization, analysis, and prediction

As such, this study intends to be a follow-up study to the work of Sircar et al. [23].

## 2. Materials and Methods

### 2.1. Materials

**Figure 1:**
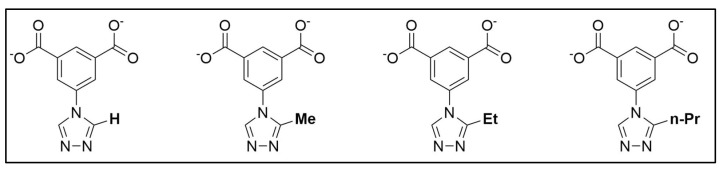
Illustration of organic linkers for the MOFs *Cu-IH-pw*, *Cu-IHMe-pw*, *Cu-IHEt-pw*, and *Cu-IHnPr-pw* from left to right. The differences are within the alkyl side-chain in the 2-position of the triazolyl ring.

As an adsorbent probing system, the MOF series composed of (Cu_2_(H-trz-Ia)_2_), (Cu_2_(H-Me-trz-Ia)_2_), (Cu_2_(H-Et-trz-Ia)_2_), and (Cu_2_(H-nPr-trz-Ia)_2_) was used as published by Kobalz et al. [26]. For better readability, the adsorbents are designated as *Cu-IH-pw*, *Cu-IHMe-pw*, *Cu-IHEt-pw*, and *Cu-IHnPr-pw* within this paper, respectively, depending on their linker structure (see Figure 1).

**Figure 2:**
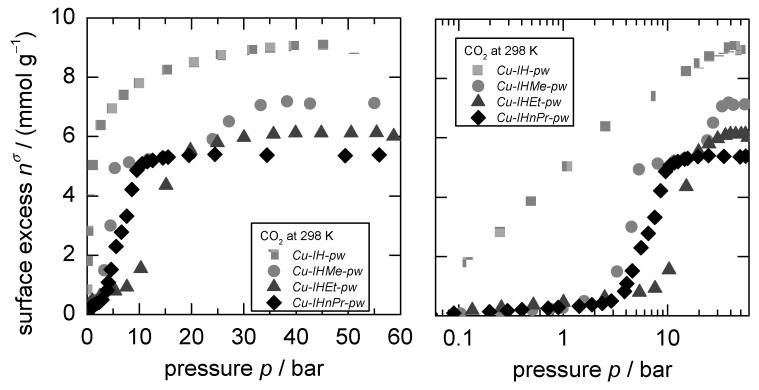
Classical adsorption isotherm in linear (**left**) and logarithmic (**right**) representation for the CO_2_-adsorption at 298 K for Cu-IH-pw, Cu-IHMe-pw, Cu-IHEt-pw, and Cu-IHnPr-pw, showing a decrease in sorptive loading in dependence of the linker size. Graphic adapted from Kobalz et al. [26].

**Figure 3:**
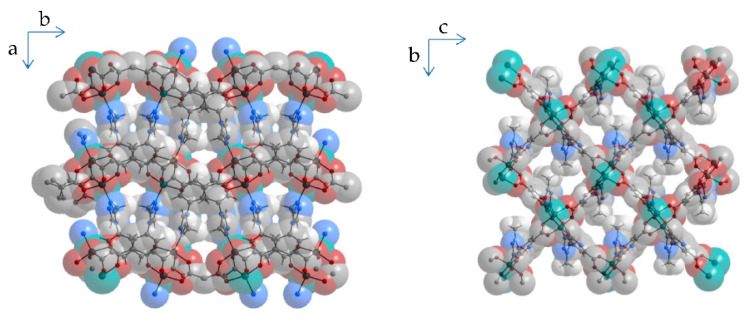
Parallel projection of *Cu-IHMe-pw* along the largest cavities in the *np* form (**left**) and the *mp* form (**right**), atoms shown with van der Waals radii.

As previously reported for CO_2_ adsorption [26], *Cu-IH-pw* shows no flexible behavior while *Cu-IHMe-pw* demonstrated two, *Cu-IHEt-pw* and *Cu-IHnPr-pw* one structural transition, making it a well-suited MOF series for the investigation of sorptive switching behavior (see Figure 2). Therefore, at least three concurring structures are existent in the flexible MOFs, herein called narrow pore, medium pore, and large pore form (*np*, *mp,* and *lp* form, respectively). For Cu-IHMe-pw, the largest cavities in the respective structure np and mp are shown in Figure 3. For further details on the structures, please see the Supplementary Materials, Section S4.

The synthesis of the MOFs was conducted according to [26]. As probing molecules, the *n*-alkanes ethane, propane, and *n*-butane, as well as the olefin *iso*-butene, were chosen in order to investigate a wide range of physical and chemical properties.

### 2.2. Methods

#### 2.2.1. Sorption Isotherms

Isotherms were measured according to a modified protocol by Keller and Staudt [27]. The adsorption and desorption isotherms of ethane, propane, and *n*-butane on the MOFs were determined in a temperature range from 283 K to 313 K and at pressures of up to 5 MPa using a magnetic suspension balance (Fa. Rubotherm GmbH, Bochum, Germany). Three pressure transducers (MKS Instruments Deutschland GmbH, Germany, Newport Omega Electronics GmbH, Germany) were used to collect data for the pressure range up to 5 MPa. Before the sorption experiments, the MOFs (0.2 g) were activated for at least 12 h at 373 K under a minimum pressure of 0.3 Pa until constant mass was achieved. Materials were used only for a maximum number of 10 cycles, which prohibits any cycling stability issues with this series of MOFs. The temperature was kept constant throughout the measurement with an accuracy of 0.5 K. Ethane, propane, and *n*-butane were obtained from Linde (Linde AG, München, Germany) with purities of 99.5%. All isotherms within this work are presented in absolute gas loading based on a buoyancy correction [27].

#### 2.2.2. Adsorption Enthalpies

To determine the adsorption enthalpy in dependence of sorptive loading, a manometric setup was coupled to a microcalorimeter. Prior to each adsorption experiment, the samples were outgassed and heated up to 333 K for 2 h. The adsorption experiments were carried out at 298 K. The heat evolved during each adsorption step was measured using a C80 microcalorimeter (Setaram, France). The heat flow to/from the sample is detected by means of 3D heat DSC sensors. The integration of heat peaks was performed by Calisto^®^ Software (v1.043 AKTS-Setaram).

#### 2.2.3. Dubinin-Based Universal Adsorption Theory (*D-UAT*)

The first connection between the well-established Dubinin theory [28] and the universal adsorption theory (UAT) from Quinn [24] was shown by Sircar et al. [23]. Within the UAT, it is assumed that two fluids with the same set of reduced parameters (1) are in corresponding states [29], basically meaning in the same position in a reduced *p-V* phase diagram. However, in terms of adsorption, the critical parameters can be used as scaling variables in order to normalize different fluid properties onto one corresponding state. In Equation (2), Dubinin’s sorption potential A is scaled via the critical temperature to a reduced adsorption potential $A_{red}$, therefore all fluid–fluid interactions are normalized. A deeper description and theoretical proof of its applicability are given in the Supplementary Materials, Section S1-II.

(1) $$T_{red}=\frac{T}{T_{C}}$$

(2) $$A_{red}=\frac{A}{T_{C}}=-R\frac{T}{T_{C}}\ln\left(\frac{p}{p_{0}}\right)=-RT_{red}\ln\left(\frac{p}{p_{0}}\right)$$

All isotherm fits in this work were performed using a dual Dubinin–Asthakov equation (dual DA, (3)) [30]. The detailed fitting approach for the accessible pore volume W in dependence of the sorption potential A or reduced sorption potential $A_{red}$, as well as the derived fitting parameters, are within the Supplementary Materials, Section S2.

(3) $$W=W_{0,1\;}e^{{\left(\frac{-A}{E_{1}}\right)}^{m_{1}}}+W_{0,2\;}e^{{\left(\frac{-A}{E_{2}}\right)}^{m_{2}}}$$

## 3. Results

### 3.1. D-UAT on the Rigid MOF Cu-IH-pw

**Figure 4:**
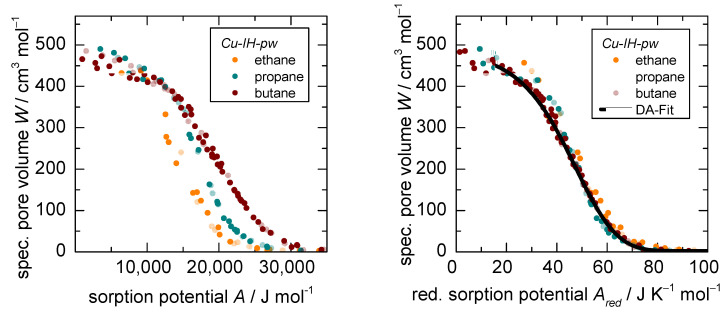
Dubinin plot before (**left**) and after (**right**) application of the Dubinin-based universal adsorption theory (*D-UAT*) ethane, propane, *n*-butane on the rigid *Cu-IH-pw* at 283 K, 298 K, and 313 K.

The Dubinin plots for ethane, propane, and *n*-butane generated from isotherms at 283, 298, and 313 K (except ethane at 313 K) on the rigid adsorbent *Cu-IH-pw* are shown in Figure 4 (left), and the classical sorption isotherms can be found in the Supplementary Materials. All sorption data points, including ad- and desorption for each sorptive, superimpose to one characteristic, temperature independent sorption pattern, thus showing no indication of a phase transition of either the fluid or the adsorbent (see also Figure S23). The overall shapes represent type Ia isotherms for microporous solids according to the IUPAC classification [31], although a continuous pore filling can be observed within a relative pressure range of 0.10–0.99 for each adsorptive. The specific accessed pore volume W reaches 480 cm^3^ per mole unit cell (with two formular units) adsorbent for all sorptives. However, as expected, the specific temperature independent characteristic sorption patterns for ethane, propane, and *n*-butane deviate in their respective positions in the Dubinin plot due to their different fluid properties. Herein, half of the sorptive loading is reached by *n*-butane at a sorption potential A of 19.5 kJ mol^−1^ and by ethane at 14.0 kJ mol^−1^.

However, when applying the *D-UAT* by dividing the sorption potential A by the critical temperature $T_{C}$ of the fluid to a reduced sorption potential $A_{red}$, all three characteristic sorption patterns coincide (Figure 4 right). This is the first time that three different gases were normalized using the *D-UAT* and a complete coincidence of the characteristic patterns is reported based on experimental data. It would furthermore underline the claim made by Sircar that a complete coincidence can be achieved with this methodology for different sorptives. The characteristic reduced sorption pattern for all three sorptives fit with a dual Dubinin–Asthakov fit (DA-fit).

**Figure 5:**
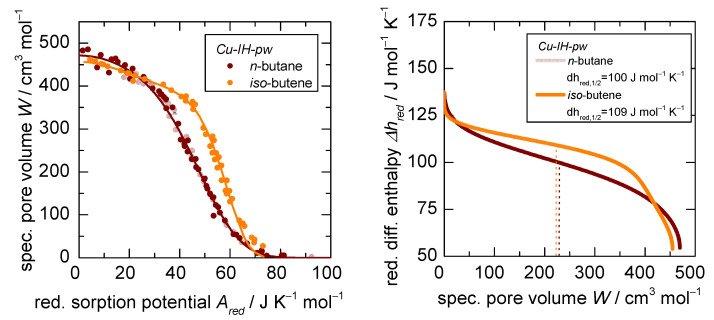
*D-UAT* plot (**left**) and reduced differential heat of adsorption $dh_{red}$ (**right**) after application of the *UAT* to the sorption of *n*-butane and *iso*-butene on *Cu-IH-pw* at 283 K, 298 K, and 313 K.

Using a chemically different adsorptive such as *iso*-butene, the reduced isotherms do not converge into the same characteristic sorption pattern, as shown in Figure 5 (left). While *iso*-butene sorption isotherms display the same overall shape and it reaches a similar total accessed pore volume W, the pattern is shifted to a higher reduced sorption potential $A_{red}$. As established in the literature, every sorption potential corresponds to a specific differential heat of adsorption [32] and thus the enthalpic interaction of solid and adsorptive is scalable with $T_{C}$ as well, if a complete coincidence of all isotherms is observed. This can be seen in Figure 5 (right), where a value of 100 ${J\;K}^{-1}{\mathrm{mol}}_{\mathrm{Gas}}^{-1}$ as reduced differential heat of adsorption $dh_{red}$ for the *n*-alkanes on *Cu-IH-pw* at half coverage can be derived via the dual Dubinin–Asthakov fit and the Clausius–Clapeyron equation [32]. For the case of a rigid adsorbent, the shift to higher reduced sorption potentials of the characteristic curve can only be explained with a higher interaction potential between solid and gas or vice versa. In the case of *iso*-butene and its double bond, it is possible that it can interact specifically with the π systems of the triazolyl or the isophtalate ring structures of the rigid MOF and thus has a higher reduced differential heat of adsorption $dh_{red}$ (108 ${J\;K}^{-1}{\mathrm{mol}}_{\mathrm{Gas}}^{-1}$ at half coverage).

Thus, by applying the *D-UAT* to rigid adsorbents, differences in the reduced sorption pattern can be assigned to two governing parameters:

1. The accessible pore volume V (derived from the modelling parameter W in Equation (3)) and
2. The reduced adsorption enthalpy $dh_{red}$ (derived from the modelling parameters E and m in Equation (3)).

Especially regarding the latter, the *D-UAT* is a practical improvement compared to the classical Dubinin theory and the empirically defined scaling parameter β [28], which has to be catalogized for every adsorbent–adsorptive system. This shows a significant difference to the work of Sircar, where it was assumed that, with the scaling of the sorption potential with the critical temperature, not only fluid–fluid but also fluid–adsorbent interactions were to be normalized. A closer look into the precise thermodynamics of adsorbed gases on rigid adsorbents under corresponding states and the mathematics regarding the *D-UAT* are given in the Supplementary Materials, Section S1-II. The methodology could also give insight into kinetic hindrances or pore-blockage effects. With the use of the dual Dubinin–Asthakov fit, one could even predict sorption isotherms of other gases such as longer *n*-alkanes on the same adsorbent. However, this requires assumptions like a neglection of size exclusion effects and always constant reduced interaction potentials, and thus, the applicability as a prediction tool should be neglected.

Therefore, the *D-UAT* can be utilized as a quick and deterministic visual and analysis tool in order to evaluate large portions of adsorption isotherm data points of chemically and physically very different gases on rigid adsorbents. So far, the theory has been used and investigated predominantly for flexible MOFs in recent years [23,33]. In the following section, the hydrocarbon sorption isotherms for the three flexible frameworks, *Cu-IHMe-pw*, *Cu-IHEt-pw*, and *Cu-IHnPr-pw*, will be examined, and the applicability of the theory for this subclass will be evaluated.

### 3.2. D-UAT on the Flexible MOFs Cu-IHMe-pw, Cu-IHEt-pw, and Cu-IHnPr-pw

**Figure 6:**
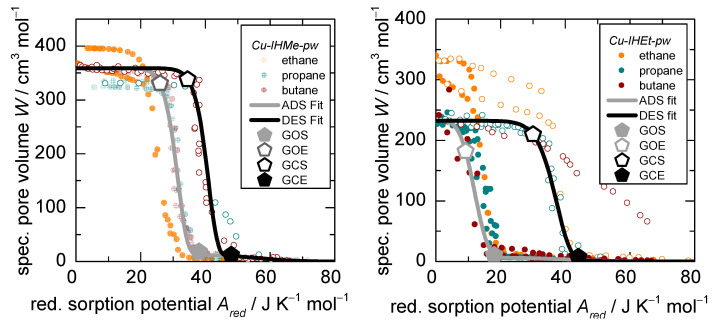
*D-UAT* plot with the reduced sorption potential *A_red_* for adsorption (filled circles) and desorption (empty circles) of C2 to C4 *n*-alkanes on the flexible *Cu-IHMe-pw* (**left**) and *Cu-IHEt-pw* (**right**). Furthermore, the respective fits of the dual Dubinin–Asthakov function are shown for both processes as well as the boundaries of the structural transition (GOS—gate opening start, GOE—gate-opening end, GCS—gate closing start, GCE—gate closing end).

The Dubinin plots after application of the *D-UAT* for ad- and desorption of ethane, propane, and *n*-butane at 283, 298, and 313 K (except ethane at 313 K) on the flexible adsorbents *Cu-IHMe-pw* and *Cu-IHEt-pw* are shown in Figure 6 (left and right, respectively).

All reduced sorption patterns show two different adsorption regimes typical for flexible MOFs. First, very low uptake at low relative pressure in a narrow pore phase (*np* phase) and, due to a structural transition, a larger medium pore phase (*mp* phase) at higher relative pressure (Note that *Cu-IHMe-pw* displays a second structural transition upon adsorption of CO_2_, as shown by Kobalz et al. [26], the then present phase is called large pore phase). Furthermore, a hysteresis is observed between ad- and desorption patterns.

Almost all isotherms shown in Figure 6 converge into characteristic patterns, although with slight deviations and generally not as sharp as seen in the case of *Cu-IH-pw* shown in Figure 2. Within *Cu-IHMe-pw*, the total accessible pore volume differences between the three sorptives are only minor. Within *Cu-IHEt-pw* (Figure 6, right), however, ethane has an increased accessible pore volume of 44% and 30%, depending on the temperature, as compared to propane and *n*-butane (340 and 300 vs. 230 cm^3^ mol^−1^). This might be due to entropic effects, given the smaller size of ethane and the ability to find a denser conformation within the pore as compared to the other sorptives. Another possibility is that ethane opens the structure to an extent that it rather resembles the large pore phase of *Cu-IHMe-pw*. The desorption of *n*-butane on *Cu-IHEt-pw* does not close the framework as opposed to ethane and propane. This may be due to a kinetic hindrance of the desorption process due to the larger molecular size of the adsorptive and a retention of *n*-butane within the MOF pores. Furthermore, the fourth MOF in the series, *Cu-IHnPr-pw*, was also probed with the *n*-alkanes but showed only adsorption within the *np* phase and thus is omitted for the analysis in this section.

In order to form a basis for a quantitative analysis, the sorption patterns were again fitted to a universal curve with the dual DA equation, where one part represents the *np* phase and the other the larger *mp* phase. Within the fitting process, the aforementioned deviations from the characteristic patterns were omitted. Furthermore, with the aid of the theory of the excess surface work (ESW) by Adolphs et al. [34], three characteristic points for both ad- and desorption, namely the gate-opening start (GOS), gate-opening end (GOE), gate-opening center (GOC), gate-closing start (GOS), gate-closing end (GCE), and gate-closing center (GCC) could be derived. All values are listed in Table 1. For the fitting approach and the ESW theory, see Supplementary Materials, Section S2.

From the universal fits, the overall accessible pore volumes per mol MOF, W, at a reduced sorption potential of 0, meaning at the respective saturation pressures, are 359 and 243 cm^3^ mol^−1^ for *Cu-IHMe-pw* and *Cu-IHEt-pw*, respectively. This marks a reduction of 25% and 50% compared to *Cu-IH-pw*, caused by the narrower pores resulting from alkyl substituents of the linkers. The accessible pore volume in the *np* phase up to the GOS, around 10 cm^3^ mol^−1^, is similar for all sorptives and both flexible hosts. This can be interpreted as adsorption only on the outer surface of the particles or within the spatial of pore entries, but no adsorption within the depths of the pore system.

Despite deviations between the characteristic patterns within the respective MOF systems, the overall convergence of all isotherms after the application of the *D-UAT* indicate similar results as seen for *Cu-IH-pw*. Published works using ethane and propane as probe molecules in other flexible MOFs [35,36] were analyzed using the same method and led to similar results, as presented in Figures S20 and S21.

For a material to be flexible, an energetic offset ($\Delta F_{}^{Host}$) between at least two concurring structures is necessary, as introduced by Coudert et al. [7]. The same logic holds true under reduced states. The calculation of this parameter from the dual DA fits is strongly dependent on whether the ad- or desorption fit is relied upon. Due to an investigated kinetic hindrance for the gate-opening process that is also seen in other works, the desorption fit is used in this paper [33,37]. A precise derivation of this logic is to be found within the Supplementary Materials, Section S1. The reduced energetic offsets based on the method of Coudert [7] resulted in 3.8 and 4.3 ${J\;X\;\mathrm{mol}}_{\mathrm{MOF}}^{-1}$ for *Cu-IHMe-pw* and *Cu-IHEt-pw*, respectively. A similar method proposed by Mason et al. resulted in similar values [10], the results are also summarized in Table 1. At 298 K, the real values of the difference of free host energy for *Cu-IHMe-pw* would be 16, 12, and 6 $J\;{\mathrm{mol}}_{\mathrm{MOF}}^{-1}$ for *n*-butane, propane, and ethane, respectively. These values are similar to those of other flexible MOFs [35,38].

Thus, by applying the *D-UAT*, differences in a reduced sorption patterns in flexible materials can be summarized by five governing parameters from three parameter clusters:

1. The accessible pore volumes V for (a) open and (b) closed form (derived from the modelling parameters $W_{0,1}$ and $W_{0,2}$ within Equation (3)),
2. The reduced adsorption enthalpies $dh_{red}$ for (a) open and (b) closed form (derived from the modelling parameters $E_{1}$/$E_{2}$ and $m_{1}$/$m_{2}$) within Equation (3) and
3. The resulting reduced energetic offset between the opened and closed structures as indicated by the calculated ($\Delta F_{red}^{Host}$, derived from all fitting parameters)

### 3.3. D-UAT Simulation and Sensitivity Analysis of Governing Parameters V, $dh_{red}^{},$ and $\Delta F_{red}^{Host}$

In order to prove the theoretical validity of the theory, a simple theoretical sorption model incorporating the van der Waals theory formulations by Johnston et al. was set up [29]. The model takes into account two concurring structures (closed pore and open pore) within one MOF with an energetic offset similar to the $\Delta F_{red}^{Host}$. Furthermore, the volume V that both structures enable for adsorption as well as the reduced heat of adsorption $dh_{red}^{}$ are relevant factors. The detailed theoretical derivation can be found in the Supplementary Materials, Section S1-IV. The three governing parameters, V, $dh_{red}^{}$, and $\Delta F_{red}^{Host}$, can thus be studied independently, enabling a complete sensitivity analysis (see Figure S7).

Within this section, the differences of the three governing parameters of the *D-UAT* between the three MOFs, *Cu-IH-pw*, *Cu-IHMe-pw*, and *Cu-IHEt-pw*, are estimated and interpreted with the aid of the model.

As shown in Table 1, the differences in reduced free host energies $\Delta F_{red}^{Host}$ between the *np* phase as well as the *mp* phase for both flexible materials, *Cu-IHMe-pw* and *Cu-IHEt-pw*, are 3.8 and 4.3 ${J\;X\;\mathrm{mol}}_{\mathrm{MOF}}^{-1},$ respectively, are rather minor (+10%). *Cu-IH-pw*, on the other hand, shows an isotherm typical for a rigid MOF. It may be possible that within this structure, a *np* phase is possible as well which, however, is not energetically favorable compared to the *mp* phase under vacuum conditions.

Thus, there are two possible explanations. First, with increasing linker size, the *mp* phase is destabilized due to a larger repulsion of the linkers. Second, the *np form* is destabilized due to stronger interactions of the linkers itself.

The first possibility was accounted for in the adsorption model in order to simulate the three MOFs and their distinct behavior. Furthermore, the specific pore volumes of the opened pore phase of *Cu-IHMe-pw* and *Cu-IHEt-pw* were reduced by 25% and 50% compared to *Cu-IH-pw*, respectively, and the closed pore phase volume was kept constant for all simulations. No energetic offset was set for *Cu-IH-pw*, while for both flexible materials this was done in accordance with the experimental data. Additionally, the reduced heat of adsorption $dh_{red}^{}$ was set as equal for all materials and pore phases. The precise calculation of this simulation can be seen in the Supplementary Materials, Section S1-V.

**Figure 7:**
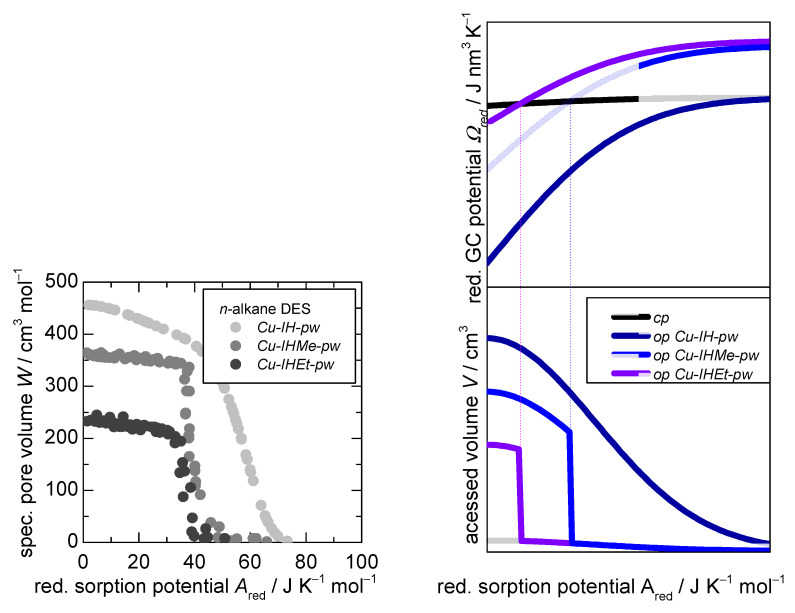
**Bottom-left**—*D-UAT* plot for the *n*-alkane desorption from experimental data at 283, 298, and 313 K. Right side: Output of the theoretical sorption model for one closed pore structure and three open pore structures in regard to the reduced grand canonical potential $\Omega_{red}$ (**top**) as well as accessed volume V (**bottom**, leading to a D-UAT plot) in dependence of the reduced sorption potential $A_{red}$. The specific inputs were chosen to reach a high resemblance between the D-UAT plot from experimental data and thus gain more insights into the material–property relationships.

The resulting reduced sorption curves are presented in Figure 7 (bottom right), showing a close resemblance of the actual system under study as in Figure 7 (bottom left). For the real-world system, there is likely a combination of the three parameters which would fully explain the differences in a *D-UAT* plot.

The theory thus aided in finding potential focus points for further research on the MOF series (Cu_2_(L-trz-Ia)_2_) in order to better understand the relationships between the *np*, *mp*, and *lp* phases and the switching behavior between them:

- Investigate a potential *np* phase for the “rigid” *Cu-IH-pw* under vacuum conditions with in situ PXRD
- Investigate whether ethane is able to open *Cu-IHEt-pw* to the *lp* phase rather than the *mp* phase utilizing in situ PXRD
- Further investigate the kinetic hindrance during the gate-opening and gate-closing processes in dependence of MOF, adsorptive, temperature, and pressure jumps
- Investigate whether the affinity of the MOF series towards olefins can be utilized for alkane–alkene separation processes.

## 4. Conclusions

In this work, it is shown that the Dubinin-based universal adsorption theory (*D-UAT*) is a quick deterministic analysis tool for the visual comparison of sorption isotherms of physically and chemically different adsorptives while being a strong quantitative tool with the aid of the Dubinin–Asthakov equation due to the reduction of differences within reduced characteristic sorption patterns to three basic features: the accessible pore volume V, the reduced enthalpy of adsorption $dh_{red}$ between host and gas and, in case of flexible materials, the reduced free host energy $\Delta F_{red}^{Host}$.

Furthermore, large datasets can be screened for anomalies or specific properties regarding these parameters or kinetic hindrances. This could accelerate the synthesis–analysis–feedback loop and enable a faster MOF research for applications like gas storage and separation without the use of computationally expensive calculations. However, the theory has limitations as the precise interplay of the accessible pore volume, reduced adsorption potential, and differences of reduced host energies can only be estimated based on at least some assumptions. The temperature dependence of $\Delta F_{red}^{Host}$ was neglected as only a small temperature range was investigated, albeit such dependency is often reported in the literature [39,40,41]. The validity of the theory for supercritical fluids is unknown and should be further investigated. Additionally, the *D-UAT* was used under the assumption that for all adsorptives, the same structural phases within one material are present with the same host energies. However, it is worth investigating whether in the case of, e.g., *iso*-butene, a structure would adjust to the different spatial demand of the adsorptive and thus different host energies would be present.

Due to the theory’s herein proven capabilities, it was possible to study three iso-reticular MOFs, two of which show flexible behavior. It could be shown that the reduced sorption patterns of the *n*-alkanes ethane, propane, and *n*-butane lead to the same results under corresponding states for all three MOFs, indicating the three governing, reduced parameters under the *D-UAT* are likely equal. The slight differences in the linkers within the MOF series have, besides the accessible pore volume, a large effect on the individual stability of the respective *mp* phases, leading to shifts of the structural transition. Further focal points for future investigations could be derived with the aid of this theory. Currently, follow-up studies focus on the mechanisms that influence the diffusion as well as the rate of structural transition for one particular MOF–gas pair, and on the complex interplay of thermodynamics and kinetics within flexible materials in more detail in order to estimate the potential of this material class for future applications.

## Figures and Tables

**Table 1:** Reduced sorption potential for gate-opening boundaries and calculated $\Delta F_{red}^{Host}$ and $\Delta H_{red}^{Host}$ values (underlined grey) of *Cu-IHMe-pw* and *Cu-IHEt-pw* taken from the characteristic dual DA fits.

Parameter	Unit	*Cu-IHMe-pw*	*Cu-IHEt-pw*
$A_{red}^{GOS}$	${J\;K}^{-1}{\;\mathrm{mol}}_{\mathrm{Fluid}}^{-1}$	38	19
$A_{red}^{GOE}$	${J\;K}^{-1}{\;\mathrm{mol}}_{\mathrm{Fluid}}^{-1}$	26	11
$A_{red}^{GOC}$	${J\;K}^{-1}{\;\mathrm{mol}}_{\mathrm{Fluid}}^{-1}$	30	14
$A_{red}^{GCS}$	${J\;K}^{-1}{\;\mathrm{mol}}_{\mathrm{Fluid}}^{-1}$	35	30
$A_{red}^{GCE}$	${J\;K}^{-1}{\;\mathrm{mol}}_{\mathrm{Fluid}}^{-1}$	44	42
$A_{red}^{GCC}$	${J\;K}^{-1}{\;\mathrm{mol}}_{\mathrm{Fluid}}^{-1}$	41	37
$\Delta F_{red}^{Host}$	${J\;X\;\mathrm{mol}}_{\mathrm{MOF}}^{-1}$	3.8	4.3
$\Delta H_{red}^{Host}$	${J\;X\;\mathrm{mol}}_{\mathrm{MOF}}^{-1}$	3.9	4.2

X herein refers to the adsorptive-specific entity $\frac{{\mathrm{cm}}^{3}}{{\mathrm{mol}}_{\mathrm{Fluid}}\;T_{c}}$ which allows an easy back calculation into the real values. More background regarding the calculation method can be found in the Supplementary Materials, Section S1.

## Data Availability

The datasets generated during and/or analyzed during the current study are available from the corresponding author.

## Supplementary Materials

It can be downloaded at: https://www.mdpi.com/article/10.3390/nano12142415/s1. The supporting material details in depth the theoretical background of the model among other additional information. The material is structured as follows: Section S1—Derivation of the Potential Theory under Corresponding States, Section S2—General fitting approach, Section S3—Derivation of equilibrium states for calculation of $\Delta F_{red}^{Host}$, Section S4—Additional Structural Data for the MOF series, Section S5—Further experimental data. experimental data. References [42,43,44,45,46,47,48,49] are cited in the supplementary materials.
