# Supplementary material for: Hydrocarbon Sorption in Flexible MOFs—Part I: Thermodynamic Analysis with the Dubinin-Based Universal Adsorption Theory (D-UAT)
Source: Nanomaterials (Basel). 2022 Jul 14;12(14):2415. doi: 10.3390/nano12142415 (PMC9317873; doi:10.3390/nano12142415)
Supplement: Supplementary file 1 [file nanomaterials-12-02415-s001.zip › nanomaterials-1770267-supplementary.pdf]

nanomaterials

Supplementary Materials

Hydrocarbon Sorption in Flexible MOFs—Part I: Thermodynamic Analysis with the Dubinin-Based Universal Adsorption Theory (*D-UAT*)

Hannes Preißler-Kurzhöfer [1,2], Marcus Lange [1], Andrei Kolesnikov [1], Jens Möllmer [1], Oliver Erhart [3], Merten Kobalz [3], Harald Krautscheid [3] and Roger Gläser [2,*]

[1] Institut für Nichtklassische Chemie e.V., Universität Leipzig, Permoserstraße 15, D-04318 Leipzig, Germany; che09bcz@studserv.uni-leipzig.de (H.P.-K.); lange@inc.uni-leipzig.de (M.L.); kolesnikov@inc.uni-leipzig.de (A.K.); moellmer@inc.uni-leipzig.de (J.M.)

[2] Institut für Technische Chemie, Fakultät für Chemie und Mineralogie, Universität Leipzig, Linnéstraße 3, D-04103 Leipzig, Germany

[3] Institut für Anorganische Chemie, Fakultät für Chemie und Mineralogie, Universität Leipzig, Johannisallee 21, D-04103 Leipzig, Germany; oliver.erhart@uni-leipzig.de (O.E.); merten.kobalz@uni-leipzig.de (M.K.); krautscheid@rz.uni-leipzig.de (H.K.)

* Correspondence: roger.glaeser@uni-leipzig.de

S1 Derivation of the Potential Theory under Corresponding States

In the following section, a general approach for the simulation of gas adsorption isotherms via a simplified model using the van der Waals theory is presented. Subsequently, the validity of the Dubinin based universal adsorption theory (*D-UAT*) for sorption in rigid materials will be proven using said model. For a different approach to proof the validity of the corresponding states theory in adsorption science, the early work of Myers, Sircar and Prausnitz [1,2] is recommended.

In the latter section, flexible materials will be considered with the modelling of two concurring structures within one adsorbent. Different scenarios will be evaluated in order to help explain differences in flexible adsorption behavior, on a qualitative basis, in the manuscript.

S1-I General approach of gas adsorption isotherm simulation via the van der Waals theory

For the description of the thermodynamics of a gas from a constant reservoir, the grand canonical potential Ω_{GC}^{g} (μ,V,T-ensemble) is commonly used. Herein, the free energy F^{g} of the gas and the chemical potential μ from the reservoir are relevant. In order to simulate an adsorbed phase in a box, one may take a mean-field approach with a potential exerted from the solid to the gas molecule (γ^{s-g}). In case of a rigid adsorbent, the thermodynamics of the solid can be neglected [3]. Note that it is an oversimplified picture of real-world systems, but it does enable an understanding of the basic mechanisms governing adsorption processes.

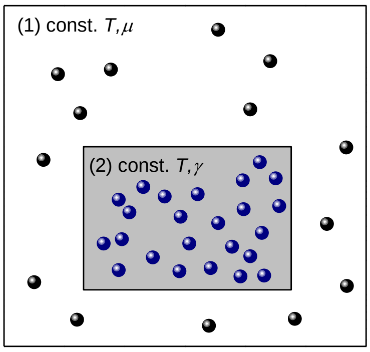

Figure S1. Schematic illustration of the adsorption model utilizing the vdW theory.

The mathematical formulation of such a system is represented in equation **Error! Reference source not found.**):

$$\Omega_{GC}^{g} = F^{g} - N\,\mu^{g} + N\,\gamma^{s-g} \tag{1}$$

where N is the number of molecules within the adsorbed system. Note that γ^{s-g} has a negative pre-sign since there are attractive forces between solid and gas. Given the van der Waals theory and its parameters a and b, the free energy and the chemical potential of the bare gas are given by the following equations which were summarized by Johnston [4]:

$$F^{g} = -N\tau\left(ln\left[\frac{n_q(V-Nb)}{N}\right]+1\right)-\frac{N^2a}{V} \tag{2}$$

The chemical potential is best derived using the fixed number density n from the gas reservoir, with $n = \frac{N}{V}$.

$$\mu^{g} = -\tau\left(ln\left[\frac{n_q(1-nb)}{n}\right]\right)+\frac{nb\tau}{1-nb}-2na \tag{3}$$

In these equations, τ stands for the thermal energy k_BT, V is the volume of the adsorption box and n_q is the quantum concentration. Inserting equations **Error! Reference source not found.** and **Error! Reference source not found.** into equation **Error! Reference source not found.**, one gets:

$$\Omega_{GC}^{g} = -N\tau\left(ln\left[\frac{(V-Nb)}{N}\right]\right)-N\tau-\frac{N^2a}{V}\mp N\tau\left(ln\left[\frac{(1-nb)}{n}\right]\right)-\frac{Nnb\tau}{1-nb}+2Nna+N\gamma^{s-g} \tag{4}$$

or:

$$\Omega_{GC}^{g} = N\,(-\tau\left(ln\left[\frac{(V-Nb)}{N}\right]\right)-\tau-\frac{Na}{V}+\tau\left(ln\left[\frac{(1-nb)}{n}\right]\right)-\frac{nb\tau}{1-nb}+2na+\gamma^{s-g}) \tag{5}$$

The last term γ^{s-g} is proportional to the measurable quantity of the differential heat of adsorption dh:

$$\gamma^{s-g} \sim dh \;\; \rightarrow N\,\gamma^{s-g} \sim dH \tag{6}$$

An illustration of the grand canonical potential can be seen in **Error! Reference source not found.**a. Under given conditions, the number of molecules within the box N_{eq}^{g} is at the minimum of the grand canonical potential Ω_{GC}^{g}, thus given by equation:

$$N_{eq}^{g} \rightarrow \frac{d\Omega_{GC}^{g}}{dN} = 0 \tag{7}$$

This minimum can be calculated for any chemical potential of the reservoir, meaning different number densities or, in more practical terms, applied pressures at constant temperatures. The resulting sorptive loading can be calculated as the first derivate of equation **Error! Reference source not found.**) to the chemical potential. Using a given isotherm, the change of the grand canonical potential can be calculated via the product of N and the change in chemical potential $d\mu^g$ as illustrated in **Error! Reference source not found.**b.

$$\frac{d\Omega_{GC}^g}{d\mu^g} = -N \quad or \quad d\Omega_{GC}^g = -N\, d\mu^g \tag{8}$$

Subsequently, a sorption isotherm of loading vs. change in chemical potential, which is mathematically the same as the negative adsorption potential in the Dubinin theory, can be calculated as illustrated in **Error! Reference source not found.**c.

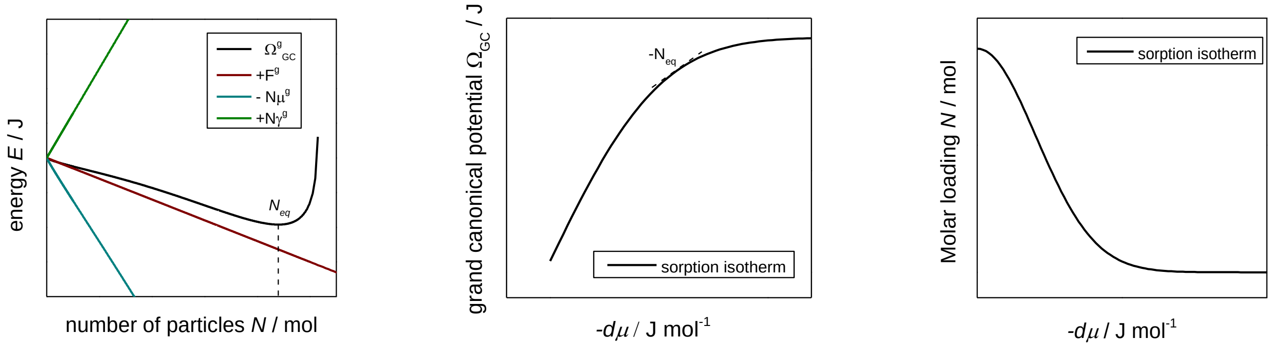

Figure S2. Visualization of the model-based adsorption isotherm construction.

Under a given temperature and pressure on one adsorbent, different gases do have different vdW parameters, leading to different chemical potentials and grand canonical potentials. Additionally, different sorption interactions with the solid may occur and thus, different adsorption isotherms are obtained (see **Error! Reference source not found.**).

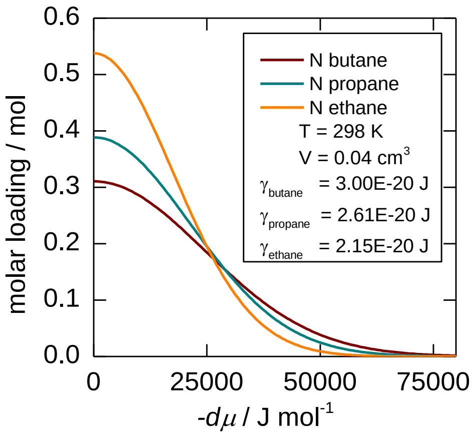

Figure S3. Visualization of different adsorption isotherms regarding ethane, propane and butane on an adsorbent with a volume of 0.4 cm^3. Furthermore, the respective interaction potentials γ^{s-g} were set arbitrary in this illustration.

S1-II Applicability of the Dubinin universal adsorption theory (D-UAT)

The objective of the bare universal adsorption theory (UAT) is to enable the normalization of gas properties via the critical temperature of chemically and physically different species and thus create more insights for the evaluation of sorptive interactions or even the prediction of adsorption isotherms from other gases based on experimental data on

one adsorbent. In addition to the bare UAT, Sircar et al. used the Dubinin theory to furthermore normalize the resulting sorptive loading to the accessed volume within an adsorbent via the adsorbate density [5]. Thus, the full application of this new theory requires two steps. One, the normalization of the independent variable in an isotherm (herein the chemical potential), and two, the normalization of the dependent variable in an isotherm, meaning the sorptive loading. To the best of our knowledge, a precise explanation or mathematical description concerning the validity of the UAT were missing so far. Thus, both steps are herein subsequently elaborated and mathematically proven. The coupling of the bare UAT (step one) and the Dubinin approach (step two) will be summarized as the D-UAT theory going forward.

In the earlier works on the UAT, the critical temperature T_c of the gas was used in order to normalize the adsorption potential A [5,6]. If the change in chemical potential $d\mu$ or adsorption potential A is scalable by T_c, then the grand canonical potential has to be scalable with T_c as well to ensure the validity of equation **Error! Reference source not found.**):

$$\frac{\Omega_{GC}}{T_c} = \int_{-\infty}^{\frac{\mu}{T_c}} -N \, \frac{d\mu}{T_c} \tag{9}$$

In order to prove the validity of the UAT it is more convenient to use the thermal energy with $k_B T = \tau$ instead of the bare temperature T, thus changing equation **Error! Reference source not found.**) to:

$$\Omega_{red}^{GC} = \frac{\Omega_{GC}}{\tau_c} = \int_{-\infty}^{\frac{\mu}{\tau_c}} -N \, \frac{d\mu}{\tau_c} \tag{10}$$

where $\Omega_{GC,R}$ is the reduced grand-canonical potential.

The van der Waals gas parameters a and b as well as the number of molecules in a system N can be expressed in terms of critical values:

$$a = \frac{27\,\tau_c^2}{64\,p_c} \tag{1}$$

$$N = \frac{8\,p_c V_c}{3\,\tau_c} \tag{2}$$

$$b = \frac{T_c}{8\,p_c} \tag{3}$$

Furthermore, critical values can be expressed in terms of the parameters a, b and N

$$p_c = \frac{a}{27\,b^2} \tag{4}$$

$$\tau_c = \frac{8\,a}{27\,b} \tag{5}$$

$$V_c = 3Nb \tag{6}$$

Reduced variables are given by the ratio of state variable and the respective critical value:

$$\tau_{red} = \frac{\tau}{\tau_R} = \frac{k_B T}{k_B T_c} \tag{7}$$

$$p_{red} = \frac{p}{p_c} \tag{8}$$

$$V_{red} = \frac{V}{V_c} \tag{9}$$

The reduced grand canonical potential, the reduced chemical potential and the reduced interaction potential are expressed as the ratio of the bare potentials and the critical thermal energy:

$$\Omega_{red}^{GC} = \frac{\Omega_{GC}}{\tau_c} \quad (10) \qquad \mu_{red} = \frac{\mu}{\tau_c} \quad (11)$$

$$\gamma_{red}^{s-g} = \frac{\gamma^{s-g}}{\tau_c} \quad (12)$$

Considering equations (1)-(9), the reduced grand canonical potential can be entirely expressed in reduced variables:

$$\frac{\Omega_{GC}}{\tau_c} = N\left(-\tau_R(\ln(3V_R - 1)) - \tau_R - \frac{9}{8V_R} + \tau_R \ln\left(\frac{3 - n_R}{n_R}\right) - \frac{\tau_R n_R}{3 - n_R} + \frac{9n_R}{4} + \gamma_R^{s-g}\right) \quad (23)$$

Under the same reduced variables, gas properties mathematically converge into corresponding states, meaning its calculation is independent of the gas used. The resulting patterns of the reduced grand canonical potential vs. the reduced chemical potential as well as the constructed isotherms can be seen on the left hand side of **Error! Reference source not found.**.

Since equation **Error! Reference source not found.** still refers to the sorptive loading as the number of molecules N, different sorptive loadings occur depending on the gases used. Thus, the second normalization step takes the parameter b, the volume of one molecule within the van der Waals theory, and multiplies with the number of molecules N to normalize the sorptive loading to the accessed volume within the adsorbate. This changes equation **Error! Reference source not found.**) to:

$$\Omega_{red}^{GC} = \frac{\Omega_{GC}\, b}{\tau_c} = \int_{-\infty}^{\frac{\mu}{\tau_c}} -Nb\, \frac{d\mu}{\tau_c} \quad (24)$$

Herein, the parameter b was used to mimic the volume of a molecule. In the real Dubinin assessment, the fluid densities in liquid state of the molecules were used, while other approaches regarding the adsorbate volume are certainly possible. Furthermore, as previously stated, only the critical temperature T_c was applied in the main manuscript instead of the thermal energy for the sake of simplicity. This leads to different units and values but does not influence the ability to analyze reduced isotherms. Note that by using the thermal energy, the x axis of the isotherms becomes dimensionless. A similar result can be obtained for the y axis when using the reduced volume with $V_R = \frac{V}{3Nb}$, thus making the reduced grand canonical potential dimensionless as well. From an experimental point of view, the accessible volume of the adsorbent is also depending on entropic effects of the adsorbent-adsorptive pair and thus would hinder a fair comparison of various isotherms with different pairs and thus was herein not applied.

The resulting isotherms from equation (24) of the adsorption model were redrawn with the dual-DA equation based on the model results and can be seen on the right hand side of **Error! Reference source not found.** for the adsorptives ethane, propane and *n*-butane. Herein, all isothermal patterns reduce to one characteristic curve. However, this only holds true since the solid-gas interaction potential γ^{s-g} are chosen to be scalable with τ_c as well, then summarized as a reduced interaction potential γ_{red}^{s-g}. As stated before, the interaction potential is proportional to the enthalpy of adsorption dh, thus the reduced interaction potential is equally proportional to the reduced adsorption potential dh_{red}.

Summarizing the chapter above, if isotherms of different gases are transformed into reduced isotherms via the herein described *D-UAT* and a complete superimposition occurs, the reduced adsorption enthalpy has to be equal for all three gases as well. For obvious reasons, the accessible volume of the adsorbent must be equal too.

To put it inversely, the shapes of isotherms reduced via *D-UAT* are determined by only two factors: first, the reduced adsorption enthalpy and second, the accessible volume of the adsorbent. Note that in this manuscript, only subcritical gases were analyzed due

to practical reasons. While in theory possible, the adsorbate density of supercritical fluids is much harder to assess and prone to large error while the independent variable would have to be changed to the absolute chemical potential instead of the change in chemical potential (requiring a saturation pressure p_0, which is not present in supercritical fluids).

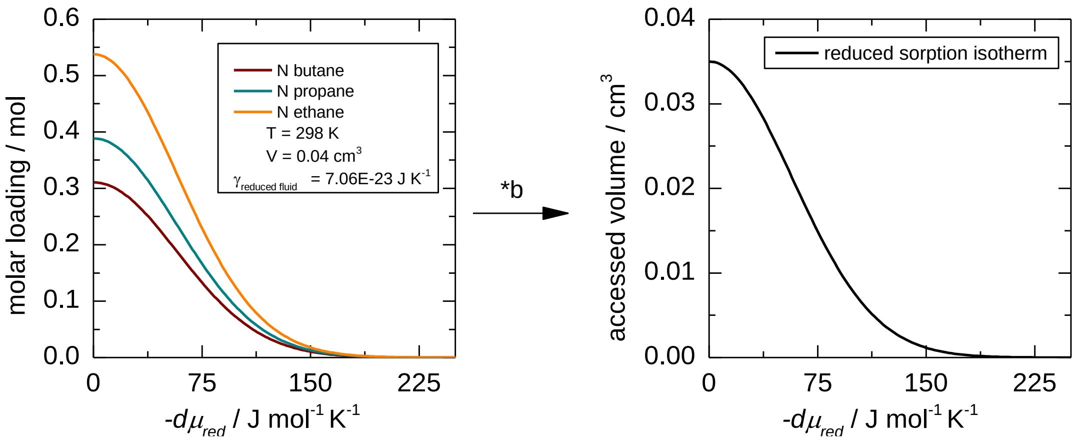

Figure S4. Illustration of the simple *UAT* (**left**) and the isotherms normalized via the adsorptive volume b further (**right**) with a Dubinin plot..

S1-III Gases in Flexible Porous Materials

A flexible porous material is present when at least two structural configurations are favorable and the energetically preferred structure changes upon change of external stimuli like pressure or temperature. The theoretic groundwork for flexible MOFs within adsorption was laid by Coudert et al. [7], showing that the position of the structural transition within an isotherm could be explained by characteristic patterns of the osmotic potential in dependence of the host´s cell volume. These patterns, also referred to as free-energy profiles, are currently a major focus of the computational chemistry community [8-10]. Using the *D-UAT*, the same thinking can be applied, an exemplary illustration is given in **Error! Reference source not found.**.

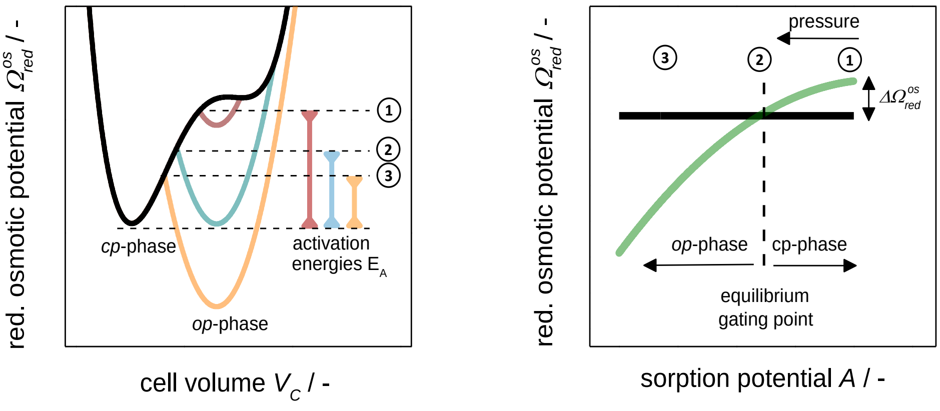

Figure S5. Schematic representation of the local and global minima of the reduced osmotic potential in dependence of the unit cell volume (**left**) as well as the resulting structural patterns for an isotherm (**right**), both under changing pressure (1-3). Herein, the red (pressure stare 1), turquois (pressure stare 2) and orange (pressure stare 3) patterns are to be understood as an extension of the black energy profile at zero pressure.

Herein, a small unit cell volume structure 1 is energetically favored at very low pressures (high reduced adsorption potentials) by an offset compared to a high unit cell volume structure 2. This offset was introduced as the energetic difference between both

empty hosts, usually denoted as dF_{Host}. With increasing pressure, thus decreasing reduced sorption potential, structure 2 becomes more favorable until a point where both structures do have the same potential under equilibrium conditions. This point would mark a sharp gate-opening, at which the loading in an isotherm switches from low to high (assuming the accessible pore volume correlates with the respective cell volume).

Thus, the adsorbent itself has to be taken into account for the thermodynamical analysis. Coudert and coworkers therefore described the adsorption in flexible hosts with the osmotic ensemble Ω_{OS}, which extends the grand canonical potential Ω_{GC}^{g} with two terms regarding the bare solid [7].

$$\Omega_{OS} = F^{s} + PV^{s} + \Omega_{GC}^{g} \tag{25}$$

In this consideration, at least two concurring host structures that are assumed as rigid are possible and only the one with the lowest osmotic potential will be present in the system, as explained in the main manuscript. Note that for a rigid material, the volume stays constant and thus, the PV^{s} term can be neglected. If the *D-UAT* theory is applied as well, one gets for both structures:

$$\frac{\Omega_{OS}\, b}{\tau_c} = \frac{F^{s}\, b}{\tau_c} + Nb\left(-\tau_R(\ln(3V_R - 1)) - \tau_R - \frac{9}{8V_R} + \tau_R \ln\left(\frac{3 - n_R}{n_R}\right) - \frac{\tau_R n_R}{3 - n_R} + \frac{9n_R}{8} + \gamma_R^{s-g}\right) \tag{26}$$

Therefore, a complete superimposition of isotherms of different gases on one flexible host in a reduced Dubinin plot means that the differential heats of adsorption are scalable by the critical temperature, and the free energy of the solid is scalable by the critical temperature and the adsorptive´s volume.

For a better illustration of this, two exemplary gas-adsorbed structures are shown in **Error! Reference source not found.** after application of the *D-UAT* in reduced quantities. One with low accessible volume and one with a 10 times larger accessible volume, however both with the same reduced differential heats of adsorption. Furthermore, it is assumed that the higher pore volume structure has an energetic offset, leading to intercrossing of Ω_{OS} for both structures and therefore to a structural transition.

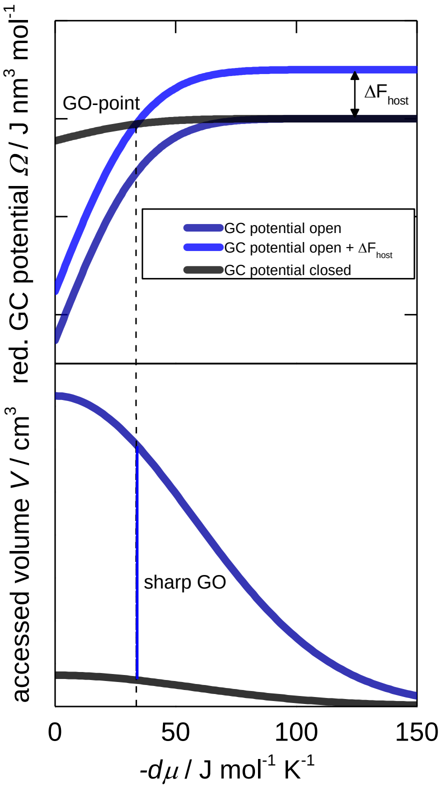

Figure S6. Reduced grand canonical potential plot vs. negative change in red. chemical potential plot for two structures with an energetic offset (ΔF_{red}^{Host}) leading to a sharp gate-opening.

S1-IV Sensitivity analysis for governing parameters $\Delta F_{red}^{Host}, V$ *and* dh_{red}

In the following, sensitivity analyses will be undertaken in order to investigate the influence of three governing parameters, $\Delta F_{red}^{Host}, V$ and dh_{red} on an opened pore phase with a constant closed pore phase. Please note that by changing any of the parameters, this would a different MOF-structure.

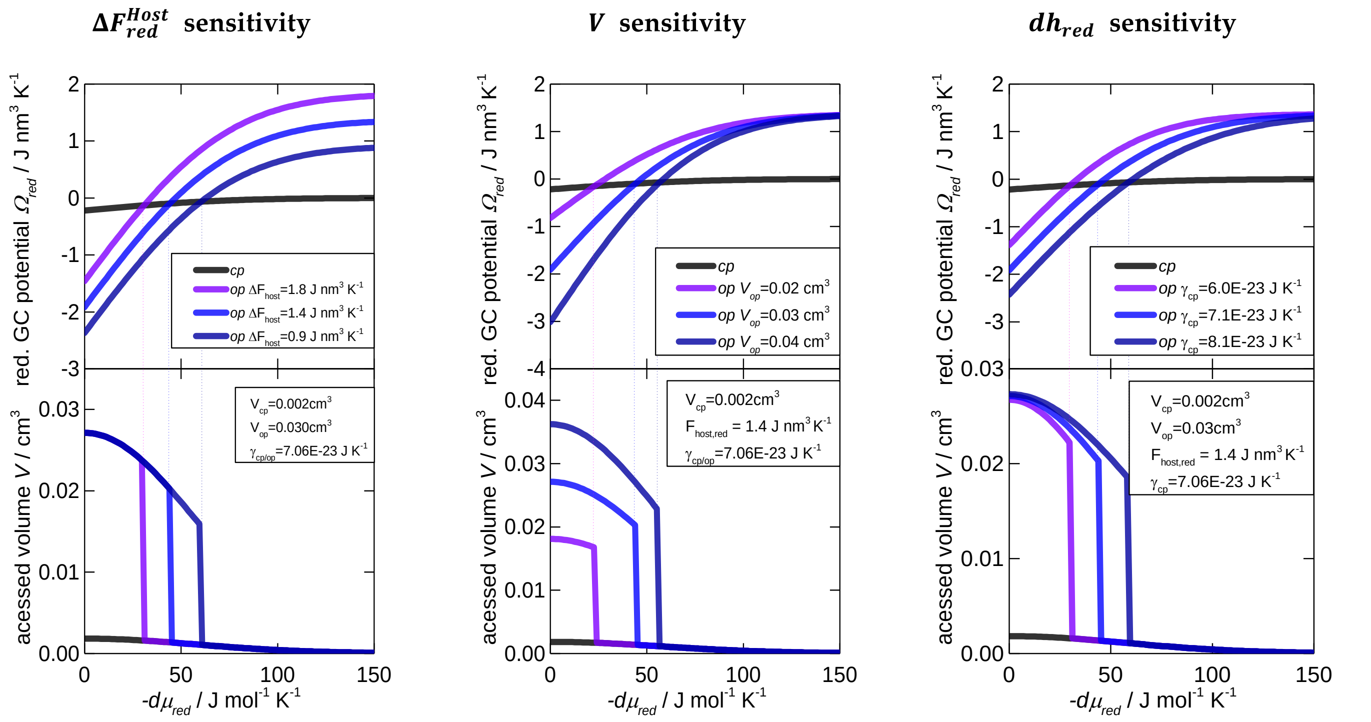

Figure S7. Sensitivity analysis regarding the three governing parameters for flexible MOFs: $\Delta F_{red}^{Host}, V$ and dh_{red}.

Error! Reference source not found. shows the dependencies of the gate-opening sorption potential. First, an increase of ΔF_{host} leads to a shift of the gate-opening point to lower reduced sorption potentials, meaning higher pressures and *vice versa* if both, V and dh_{red}, are kept constant. Secondly, a larger pore volume for the opened phase also leads to lower reduced sorption potentials since more sorption and thus more energy can be converted into the structure. Lastly, an increase of the reduced enthalpy of sorption leads to lower reduced sorption potentials for the structural transition for the same reason.

S1-V Simulation of real cases as in Cu-IH-pw, Cu-IHMe-pw and Cu-IHEt-pw

In the following, the key differences that govern the flexible behavior, or lack thereof, for *Cu-IH-pw, Cu-IHMe-pw* and *Cu-IHEt-pw* are implemented within the described model. Herein, the spec. pore volumes of the opened pore phase of *Cu-IHMe-pw* and *Cu-IHEt-pw* are reduced by 25% and 50% compared to *Cu-IH-pw*, respectively (0.04, 0.03 and 0.02 cm^3). The closed pore phases were kept constant for all materials at V_{cp} = 0.002 cm^3. Furthermore, no energetic offset was set for *Cu-IH-pw* while for both flexible materials this was implemented with 1.4 and 1.5 J nm^3 K^{-1} for *Cu-IHMe-pw* and *Cu-IHEt-pw*, respectively. Additionally, the reduced interaction potential was set equal for all materials and pore phases. The result can be seen in **Error! Reference source not found.**, showing very similar trends as compared to the real experimental data. The *Cu-IH-pw* emulation shows obviously no flexible behavior due to the missing energetic offset. The gate-opening for *Cu-IHMe-pw* occurs at a reduced gate-opening potential of 47 J mol^{-1} K^{-1}, whilst for *Cu-IHEt-pw* at 18 J mol^{-1} K^{-1}.

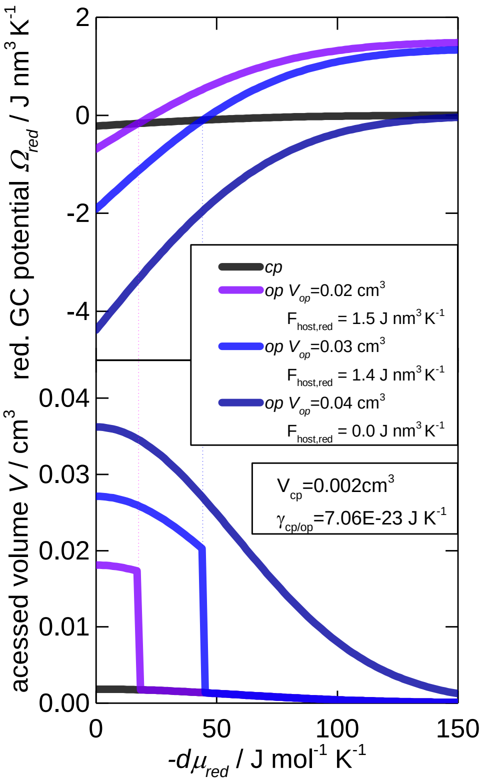

Figure S8. Simulation of the flexible behavior of the three MOFs under study with varying governing parameters of the described adsorption model.

S2 Isotherm Fitting for flexible MOFs

Within this work, a dual-Dubinin-Asthakov equation (dual-DA) suitable for inhomogeneous adsorbents with two adsorption spaces was applied to model all experimental isotherms [11].

$$W = W_{0,1}\, e^{\left(\frac{-A}{E_1}\right)^{m_1}} + W_{0,2}\, e^{\left(\frac{-A}{E_2}\right)^{m_2}} \tag{27}$$

Herein, W_0 is the saturation loading, while E and m mark the specific adsorption energy and the inhomogeneity parameter respectively. Since it is a dual-volume model, all three parameters are denoted with either 1 or 2 for a closed and opened pore phase, respectively. The fits for the n-butane adsorption on **Cu-IHMe-pw** can be observed in **Error! Reference source not found.**, the fitting parameters are presented in **Error! Reference source not found.**.

After fitting, essential points within the isotherms can be specified. For flexible MOFs, it is common that one gate-opening pressure is defined. However, due to particle size distributions of the adsorbents, different energy barriers for the structural transition can occur and therefore, pressure ranges rather than isobaric phase changes are observed. Thus, in this work, two pressure points are defined to describe the entire gate-opening. For flexible MOFs, the first term in the dual-DA equation 27 describes the sorption in the narrow pore phase (*np* phase), while the second term describes the structural transition and medium pore phase (*mp* phase) sorption. The gate-opening starts when the latter term starts adding significant pore volume. Therefore, to find a gate-opening start (GOS) point in the isotherm, a boundary value of 0.01 $cm^3\ g^{-1}$ for the *mp* phase term was set.

Secondly, a gate-opening end (GOE) is determined, herein defined using the excess surface work (ESW). The ESW, usually computed as the product of the adsorbed amount and the change in chemical potential, gives rise to the strength of interaction between the sorbent and the sorptive according to Adolphs [12]. It is mathematically identical to the first derivative of the surface potential as defined by Myers [3]. It describes the counter acting processes of decreasing surface free energy and increasing isothermal and isobaric work of sorption. The occurring minimum in the plots can then be identified as the completion of a monolayer [12].

In this work, the product of the adsorption potential and the volumetric loading was used to identify the position of a quasi-monolayer at an ESW maximum (see **Error! Reference source not found.**). It has to be stated that the resulting ESW values are not of relevance in this case but just the position of the extrema. The resulting values for the GOS and GOE points are summarized in **Error! Reference source not found.**.

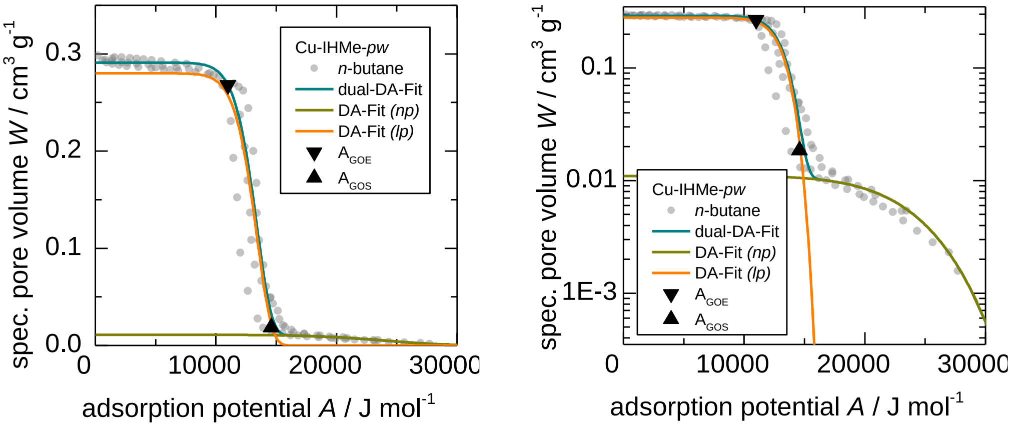

Figure S9. Fitting approach using the dual-DA equation for *n*-butane on *Cu-IHMe-pw* and the resulting gate-opening boundaries. **(left**: linear, **right**: logarithmic scale**).**

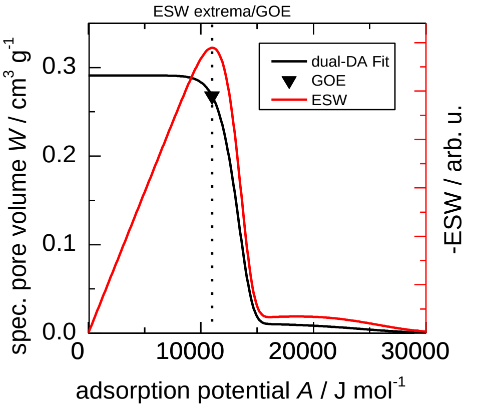

Figure S10. Determination of gate-opening end using the excess surface work (ESW) theory.

Table 1. Reduced fitting parameters for the dual-Dubinin-Asthakov model regarding the desorption of *n*-alkanes.

	**Pore phase *i***	**$W_{0,i}$ / cm³ g⁻¹**	**E_i / J K⁻¹ mol⁻¹**	**n_i**
Cu-IH-pw	2 (medium pore phase m*p*)	0.40	−52	4
Cu-IHMe-pw	1 (narrow pore phase *np*)	0.011	−59	6
	2 (medium pore phase m*p*)	0.28	−41	20
Cu-IHEt-pw	1 (narrow pore phase *np*)	0.005	−41	4
	2 (medium pore phase m*p*)	0.175	−38	10

S3 Derivation of Equilibrium states for calculation of ΔF_{red}^{Host}

As explained in the main manuscript, the calculation of ΔF_{red}^{Host} as proposed by Coudert et al. [7] is highly dependent on whether the ad- or desorption is taken into account. Thus, there stands the questions which curve represents the equilibrium state the most. In the following section, this question is being tackled from both the kinetic as well as the microcalorimetric point of view.

S3-I Kinetic analysis

To answer this question, the respective gate-opening and gate-closing ranges in both hosts can be taken as an indication. E.g. in *Cu-IHMe-pw*, ΔF_{red}^{Host} = 38-26 J mol^{-1} K^{-1} for gate-opening, 35-44 J mol^{-1} K^{-1} for gate-closing), the ranges do correspond to much tighter pressure ranges regarding the desorption as compared to the adsorption (given *n*-butane at 298 K, the gate-opening refers to pressures between 6 and 25 mbar, gate-closing to 7 and 1 mbar; note that the sorption potential is logarithmic in nature, thus bare sorption potential ranges cannot be compared in that manner). Since the pressure ranges are predominantly ascribed to kinetic hindrances of the underlying processes [13], it indicates a larger kinetic control for the gate-opening as compared to the gate-closing. This can be further substantiated by kinetic uptakes taken from the equilibrium measurements on *Cu-IHMe-pw* and *Cu-IHEt-pw*, as illustrated in **Error! Reference source not found.**. Herein, the time to reach quasi-equilibrium is much shorter for the gate-closing as compared to the gate-opening.

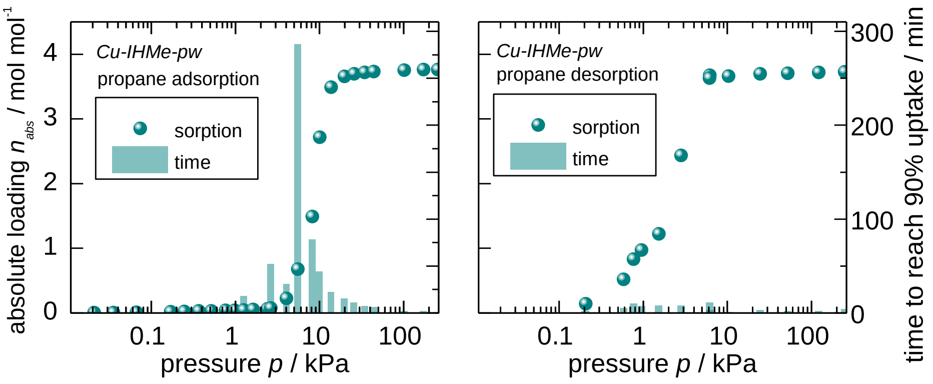

Figure S11. Visualization of the simplified kinetic analysis of adsorption (**left**) and desorption (**right**) for propane on *Cu-IHMe-pw*. Herein, the measured time to reach a quasi-equilibrium is represented as vertical bars.

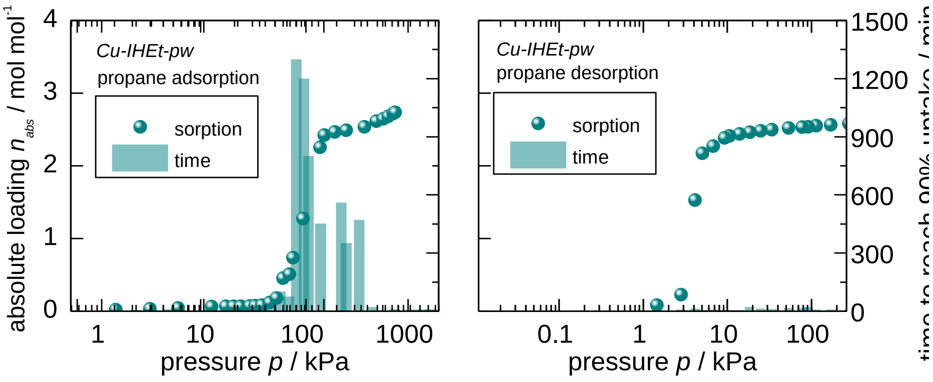

Figure S12. Visualization of the simplified kinetic analysis of adsorption (**left**) and desorption (**right**) for propane on *Cu-IHEt-pw*. Herein, the measured time to reach a quasi-equilibrium is represented as vertical bars.

Using the free-energy profile analogy from **Error! Reference source not found.**, both energetic minima regarding the respective structures 1 and 2 have the same energy at the point of structural transition (pressure point 2). Thus, the system is equally likely to "jump over" the needed activation energy going from structure 1 to structure 2 as vice versa if taken schematics of the transition state theory (TST) into account [14]. Therefore, the bare kinetics of the processes should have the same rate at the equilibrium point. However, within the gate-opening process, the gas molecules do have to enter the pore entries of the *np* phase. Additionally, there must be a minimum amount of molecules within a closed crystal domain in order to infuse the needed structural stress and subsequently trigger the transition. Conversely, during the gate-closing process, gas molecules must only leave the open host via the much wider *mp* phase. Furthermore, it is possible that the subsequent collapse of the structure would accelerate the diffusion during the desorption process as molecules are "pushed" out of the crystal domains (compare **Error! Reference source not found.**). However, the precise mechanism governing the dynamic process during structural transitions are beyond the scope of this article and will be addressed in detail in future works for the underlying system.

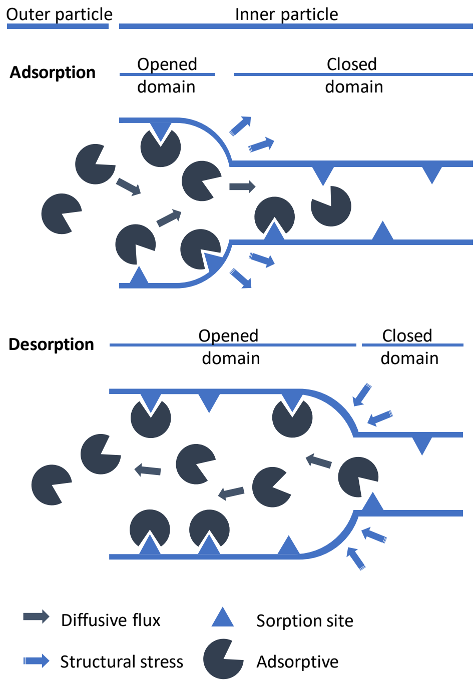

Figure S13. Schematic representation of the governing processes during ad- and desorption within flexible MOFs.

The fact that the kinetic hindrance is far more pronounced during the adsorption is also in line with the respective position of the process centers for both structures. For *Cu-IHMe-pw* and *Cu-IHEt-pw*, the GOC diverge much more (ΔF_{red}^{Host} = 30 vs. 14 J mol^{-1} K^{-1}) than regarding the GCC (41 vs 37 J mol^{-1} K^{-1}), most likely due to the much-hindered diffusion of the gas molecules in the tighter pore system of *Cu-IHEt-pw*. This is further visualized in **Error! Reference source not found.**, where the *n*-butane adsorption on *Cu-IH-pw*, *Cu-IHMe-pw*, *Cu-IHEt-pw* as well as on *Cu-IHnPr-pw*, is shown. Herein, it becomes observable that the starting potentials of the gate-opening are shifted to higher pressures the larger the alkyl substituent of the linker becomes, up to the point where no structural transition can be observed anymore in case of *Cu-IHnPr-pw*. Herein, the pore channels are likely to tight in order to enable an effective diffusion.

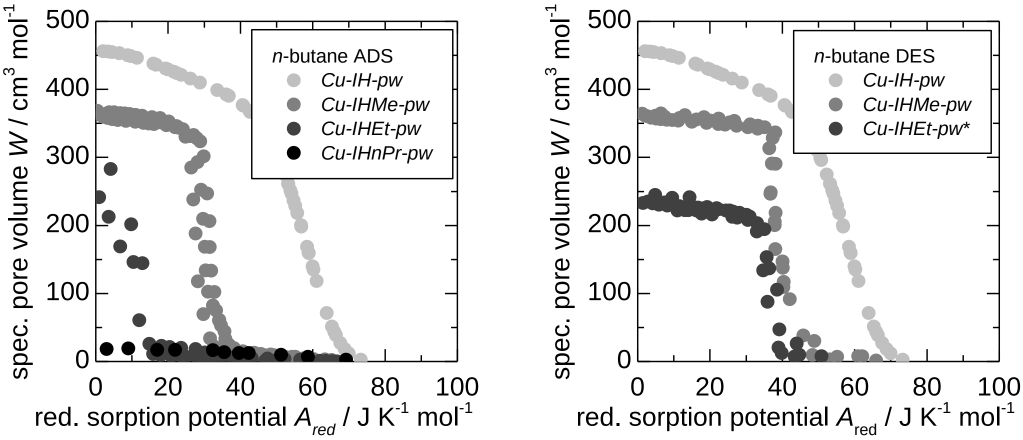

Figure S14. Reduced ad and desorption pattern of *n*-butane in *Cu-IH-pw, Cu-IHMe-pw, Cu-IHEt-pw* and *Cu-IHnPr-pw*. *Note that due to the retention of *n*-butane within *Cu-IHEt-pw* during desorption, the pattern of propane is shown.

The indications above lead to the consideration that the desorption patterns represent the thermodynamic equilibrium during the structural transition much more closely than the adsorption. This should be equally true for similar MOF systems. Using his Monte Carlo simulation tool, Numaguchi comes to the same result [14] while the kinetic differences between ad- and desorption were so far not analyzed experimentally, to the best of our knowledge.

Thus, the calculations regarding the respective energetic offsets between the *np* and *mp* phase in the MOFs under study were based on the desorption patterns. The estimation of the difference of the free host energy under the *D-UAT*, ΔF_{red}^{Host}, can be conducted in a similar way as proposed by Coudert et al. for the MIL series [7]. It relies on the integration of the hypothetically rigid sorption isotherms for the *np* phase and *mp* phase until the center of structural transition. The sorption isotherm of the *np* phase can be taken from the observable adsorption within that phase at low pressures and can be extrapolated to the saturation pressure using a single Dubinin-Astakhov fit. The hypothetical rigid isotherm of the *mp* phase poses a definite challenge for a precise quantification since this is mostly unknown and thus source of potential errors [7]. Within this work, the dDA fit of the rigid and isoreticular *Cu-IH-pw* was used and scaled to the accessible pore volumes of both *Cu-IHMe-pw* and *Cu-IHEt-pw* in order to circumvent this problem. This allows the integration of the reduced patterns and yields a universal, non-gas specific ΔF_{red}^{Host} with the unit of $J\ mol^{-1}\ X$. Herein, X stands for the gas and temperature specific molar density as well as the critical temperature of the gas used $(\frac{cm^3}{mol_{Fluid}\,T_c})$, these allow an easy recalculation to the real values. This further answers the question of whether the unreduced ΔF^{Host} should be the same for all adsorptives under the assumption of the very same opened pore structure [15,16]. Since both the molar density and the critical temperature are taken into account, different values of the ΔF^{Host} in real values have to occur (compare **Error! Reference source not found.**). The universal values for ΔF_{red}^{Host} are tabulated in **Error! Reference source not found.** as well. At 298 K, the real values of the difference of free host energy for *Cu-IHMe-pw* would be ΔF_{red}^{Host} = 16, 12 and 6 $J\ mol_{MOF}^{-1}$ for *n*-butane, propane and ethane, respectively (see **Error! Reference source not found.**). These values are comparable to other flexible MOFs [15,16].

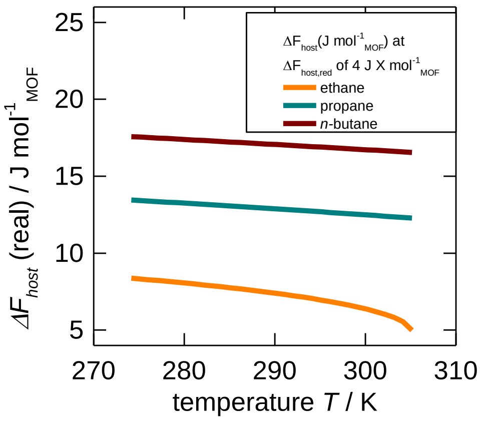

Figure S15. Temperature and gas dependence of the ΔF_{host} under real values (in $J\ mol_{MOF}^{-1}$) assuming a ΔF_{MOF}^{R} of 4 $J\ X\ mol_{MOF}^{-1}$.

S3-II Microcalorimetric analysis

Another way to derive an energetic difference between the two phases is the calculation of loss of enthalpy due to the structural transition in relation to a rigid host. **Error! Reference source not found.** shows the calculated curve for the differential enthalpy of adsorption for *Cu-IHMe-pw* and *n*-butane at 298 K. Additionally, results for this quantity derived from microcalorimetry experiments are in good agreement with the calculation. Both patterns show a drastic drop of the differential heat of adsorption during the structural transition. This was previously observed by Mason et al. for the MOF Co(bdp) [17]. The authors linked this drop directly to the amount of enthalpy that has to be put into the system for the transition to occur. Furthermore, a calculation method was proposed which similarly to Coudert´s method relies on the hypothetical isotherm for the larger pore phase [17]. The results for this method regarding the MOFs ΔH_{red}^{Host} under study herein are presented in **Error! Reference source not found.** as well, generally showing the same trend as ΔF_{red}^{Host}.

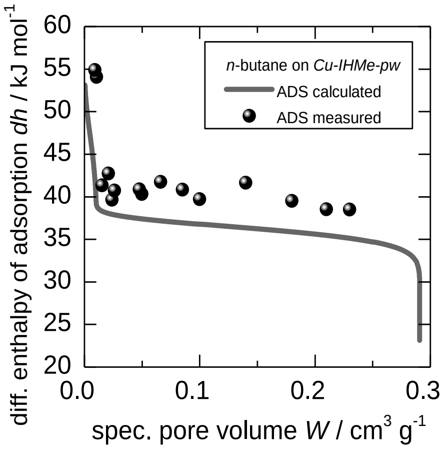

Figure S16. Calculated and measured differential enthalpy of adsorption *dh* for the *n*-butane adsorption on *Cu-IHMe-pw*.

S4 Further Structure Information

The crystallographic data is taken from Kobalz et al. [18] and the PhD Thesis of Merten Kobalz at the Universität Leipzig [19] and is summarized in **Error! Reference source not found.**.

Table 2. Crystallographic data of *Cu-IH-pw, Cu-IHMe-pw* and *Cu-IHEt-pw*.

**Compound**<br>**Form**	**[$Cu_2(H_2$-trz-Ia$)_2$]**<br>**Opended**	**[Cu_2(H-Me-trz-Ia$)_2$]**<br>**Opened *mp* form**	**[Cu_2(H-Me-trz-Ia$)_2$]**<br>**Closed *np* form**
Analysis method	single crystal data	crystal powder refinement	crystal powder refinement
Molar Mass UC / g mol^{-1}	1178.84	1234.94	1234.94
Crystal system	monoclinic	monoclinic	monoclinic
Space group	*P* 2_1/c (no. 14)	*P* 2_1/n (no. 14)	*P* 2_1/c (no. 14)
Unit cell parameters / pm, °	a = 1096.8(2))<br>b = 1220.3(1)<br>c = 1436.6(2)<br>β = 110.30(1)	a = 1135.94(5)<br>b = 1498.83(5)<br>c = 1098.10(5)<br>β = 116.582(3)	a = 1022.49(5)<br>b = 1522.16(6)<br>c = 775.14(4)<br>β = 107.792(5)
Volume / 10^6 pm^3	1803.3(5)	1672.0(1)	1148.7(1)
Z	2	2	2
Density / g cm^3	1.086	1.457	1.785
Porosity / %	52.6	43.3	4
Pore Volume / cm^3 g^{-1}	0.484	0.294	0.023
R_{int}			
R_1			
wR_2			
R_p		0.0668	0.0607
R_{wp}		0.0858	0.0750

* Structure indication of *Cu-IHEt-pw* was based on the large pore form of *Cu-IHMe-pw* and thus cannot justifingly compared.

Structure determination of *Cu-IHMe-pw* (*mp* form) and (*np* form) from X-ray powder diffraction data

High resolution X-ray powder diffraction (PXRD) data were collected on a STOE STADI-P diffractometer using Cu-$K_{\alpha 1}$ radiation (λ = 154.060 pm). For the measurements an *in situ* PXRD/Sorption setup [20] was used. The powder pattern of the *np* form was collected under vacuum, while the pattern of the *mp* form was collected in *n*-butane gas at a pressure of 500 mbar.

Indexing of the powder diffraction patterns, space group determination and structure solution was performed with EXPO2014 . Appropriate structure models could be obtained in space groups $P2_1/c$ (*np* form) and $P2_1/n$ (*mp* form) with the simulated annealing approach. One linker molecule and one Cu^{2+} ion were provided as structure fragments, respectively. In the case of the *mp* form the addition of a n-butane molecule was necessary for a successful structure solution. Subsequently, the obtained structure models were refined via the Rietveld method using TOPAS [21] and GSAS [22].

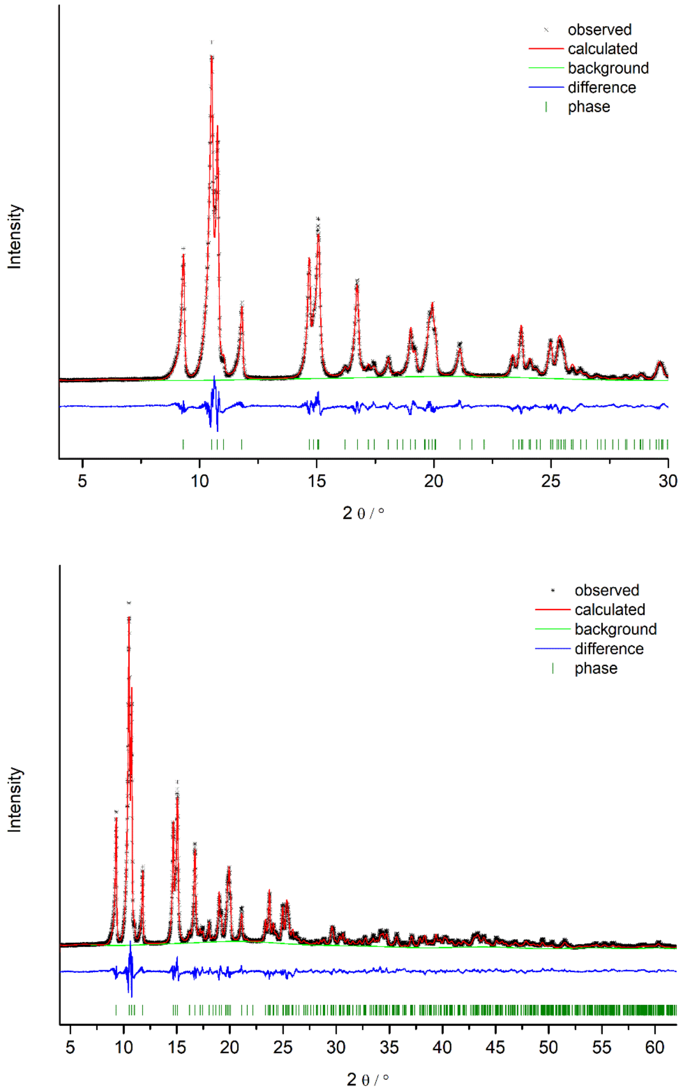

Figure S17. Rietveld refinement of *Cu-IHMe-pw* (*mp* form): Space group $P2_1/n$ (no. 14); a = 1135.94(5) pm, b = 1498.83(5) pm, c = 1098.10(5) pm, β = 116.582(3)°, Z = 2, V = 1672.0(1) 10^6 pm^3; R_p = 0.0694, R_{wp} = 0.0896.

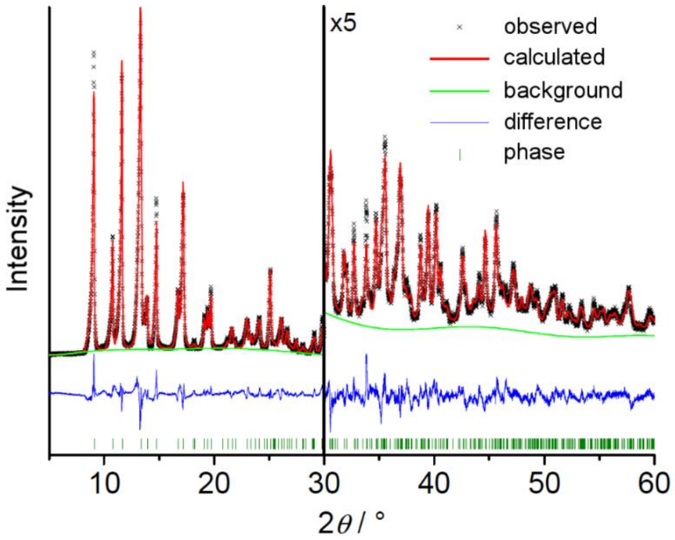

Figure S18. Rietveld refinement of *Cu-IHMe-pw* (*np*-form): *P*21/*c* (no. 14); a = 1022.49(5) pm, 1522.16(6) pm, 775.14(4) pm, β = 107.792(5), Z = 2, V = 1148.7(1) 10^6 pm^3; R_p = 0.0607, R_{wp} = 0.0750.

S5 Further experimental data

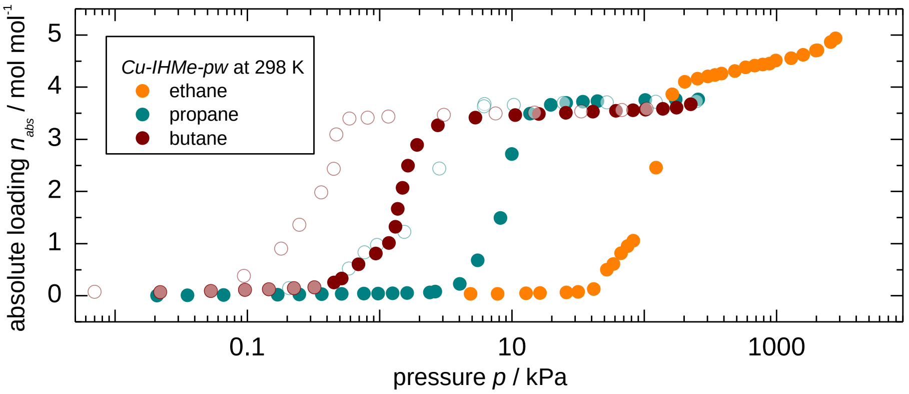

Figure S19. Ad- and desorption isotherms of alkanes at 298 K on the flexible *Cu-IHMe-pw* (adsorption - filled symbols, desorption - empty symbols).

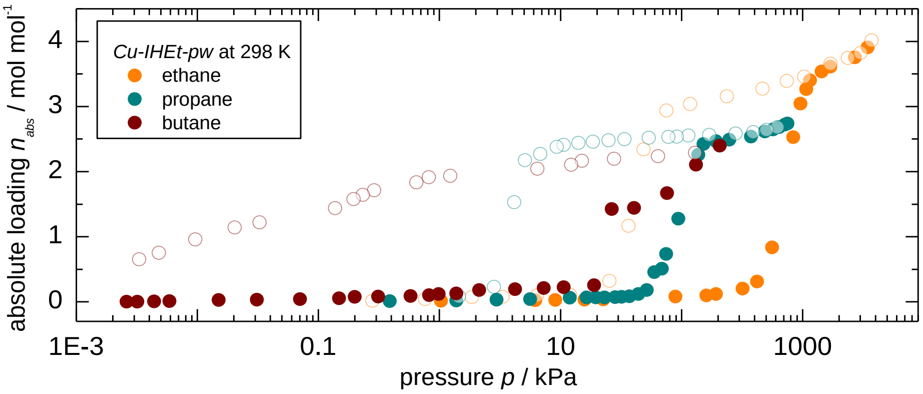

Figure S20. Ad- and desorption isotherms of alkanes at 298 K on the flexible *Cu-IHEt-pw* (adsorption - filled symbols, desorption - empty symbols).

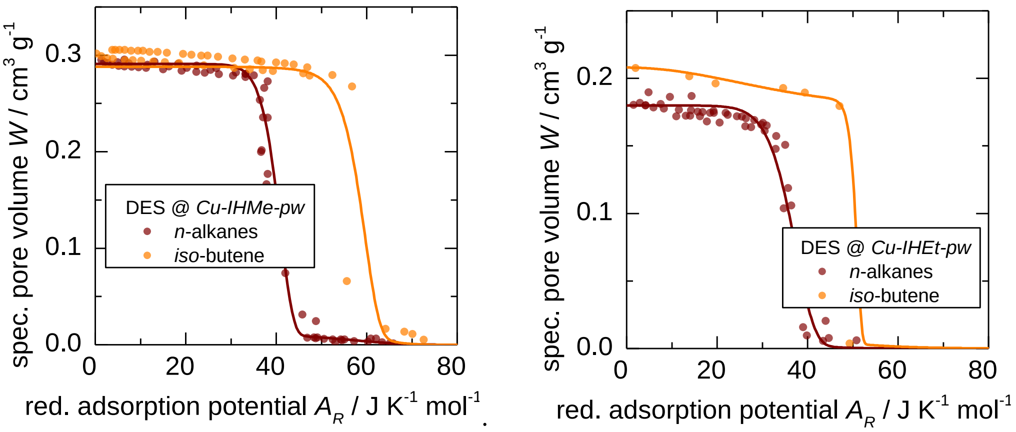

Figure S21. Dubinin plots after application of *D-UAT* to the sorption of *n*-butane and *1*-butene on *Cu-IHMe-pw (top)* and *Cu-IHEt-pw (bottom)*.

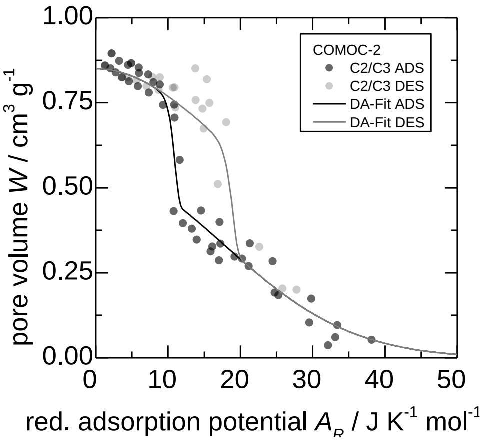

Figure S22. Dubinin plot for ad- (dark grey dots) and desorption (light grey dots) after application of *D-UAT* to the sorption of propane and ethane on Comoc-2 [15].

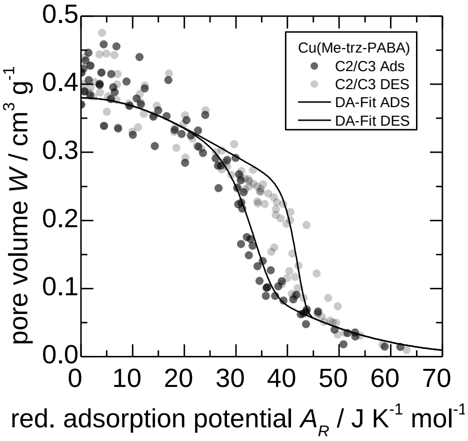

Figure S23. Dubinin plot ad- (dark grey dots) and desorption (light grey dots) after application of *D-UAT* to the sorption of propane and ethane on Cu-Me-trz-PABA [23].

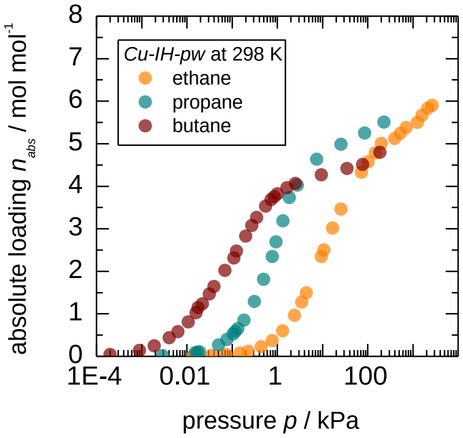

Figure S24. Adsorption isotherms of *n*-alkanes on the rigid MOF *Cu-IH-pw* at 298 K.

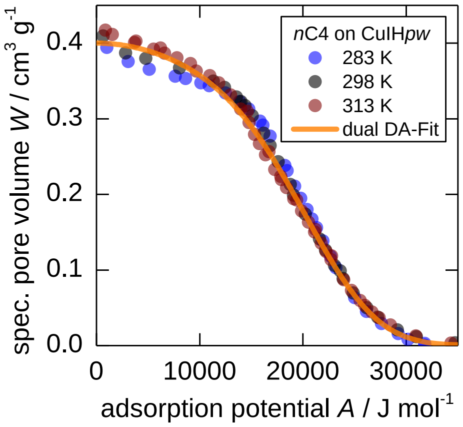

Figure S25. Dubinin plot for the adsorption of *n*-butane at 283, 298 and 313 K on *Cu-IH-pw*. The experimental data is fitted with the dual-Dubinin-Asthakov (dual-DA) model.

S6 References ESI

[1] Sircar, S.; Myers, A.L. Characteristic adsorption isotherm for adsorption of vapors on heterogeneous adsorbents. *AIChE J.* 1986, *32*, 650–656.

[2] Myers, A.L.; Prausnitz, J.M. Prediction of the adsorption isotherm by the principle of corresponding states. *Chem. Eng. Sci.* **1965,** *20,* 549–556.

[3] Myers, A.L. Thermodynamics of adsorption in porous materials. *AIChE J.* **2002**, *48*, 145–160

[4] Johnston, D.C. Thermodynamic properties of the van der Waals fluid. In *Advances in Thermodynamics of the van der Waals Fluid*; Morgan&Claypool: San Rafael, CA, 2014.

[5] Sircar, S.; Pramanik, S.; Li, J.; Cole, M.W.; Lueking, A.D. Corresponding states interpretation of adsorption in gate-opening metal-organic framework Cu(dhbc)$_2$(4,4′-bpy). *J. Colloid Interface Sci.* **2015**, *446*, 177–184. https://doi.org/10.1016/j.jcis.2015.01.011.

[6] Quinn, D.F. Supercritical adsorption of 'permanent' gases under corresponding states on various carbons. *Carbon* **2002**, *40*, 2767–2773. https://doi.org/10.1016/S0008-6223(02)00200-2.

[7] Coudert, F.-X.; Jeffroy, M.; Fuchs, A.H.; Boutin, A.; Mellot-Draznieks, C. Thermodynamics of guest-induced structural transitions in hybrid organic-inorganic frameworks. *J. Am. Chem. Soc.* **2008**, *130*, 14294–14302. https://doi.org/10.1021/ja805129c.

[8] Tanaka, H.; Miyahara, M.T. Free energy calculations for adsorption-induced deformation of flexible metal–organic frameworks. *Curr. Opin. Chem. Eng.* **2019**, *24*, 19–25. https://doi.org/10.1016/j.coche.2019.01.001.

[9] Vanduyfhuys, L.; Rogge, S.M.J.; Wieme, J.; Vandenbrande, S.; Maurin, G.; Waroquier, M.; van Speybroeck, V. Thermodynamic insight into stimuli-responsive behaviour of soft porous crystals. *Nat. Commun.* **2018**, *9*, 204. https://doi.org/10.1038/s41467-017-02666-y.

[10] Ghysels, A.; Vanduyfhuys, L.; Vandichel, M.; Waroquier, M.; van Speybroeck, V.; Smit, B. On the thermodynamics of framework breathing: A Free Energy Model for Gas Adsorption in MIL-53. *J. Phys. Chem. C* **2013**, *117*, 11540–11554. https://doi.org/10.1021/jp311601q.

[11] Burevski, D. The application of the Dubinin-Astakhov equation to the characterization of microporous carbons. *Colloid Polym. Sci.* **1982**, *260*, 623–627. https://doi.org/10.1007/BF01422595.

[12] Adolphs, J. Excess surface work?: A modelless way of getting surface energies and specific surface areas directly from sorption isotherms. *App. Surf. Sci.* **2007**, *253*, 5645–5649. https://doi.org/10.1016/j.apsusc.2006.12.089.

[13] Sircar, S.; Wu, H.; Li, J.; Lueking, A.D. Effect of time, temperature, and kinetics on the hysteretic adsorption-desorption of H2, Ar, and N2 in the metal-organic framework Zn2(bpdc)2(bpee). *Langmuir* **2011**, *27*, 14169–14179. https://doi.org/10.1021/la202842m.

[14] Numaguchi, R.; Tanaka, H.; Watanabe, S.; Miyahara, M.T. Simulation study for adsorption-induced structural transition in stacked-layer porous coordination polymers: Equilibrium and hysteretic adsorption behaviors. *J. Chem. Phys.* **2013**, *138*, 54708.

[15] Couck, S.; van Assche, T.R.C.; Liu, Y.-Y.; Baron, G.V.; van der Voort, P.; Denayer, J.F.M. Adsorption and separation of small hydrocarbons on the flexible, vanadium-containing MOF, COMOC-2. *Langmuir* **2015**, *31*, 5063–5070. https://doi.org/10.1021/acs.langmuir.5b00655.

[16] Bousquet, D.; Coudert, F.-X.; Fossati, A.G.J.; Neimark, A.V.; Fuchs, A.H.; Boutin, A. Adsorption induced transitions in soft porous crystals: An osmotic potential approach to multistability and intermediate structures. *J. Chem. Phys.* **2013**, *138*, 174706. https://doi.org/10.1063/1.4802888.

[17] Mason, J.A.; Oktawiec, J.; Taylor, M.K.; Hudson, M.R.; Rodriguez, J.; Bachman, J.E.; Gonzalez, M.I.; Cervellino, A.; Guagliardi, A.; Brown, C.M.; et al. Methane storage in flexible metal-organic frameworks with intrinsic thermal management. *Nature* **2015**, *527*, 357–361. https://doi.org/10.1038/nature15732.

[18] Kobalz, M.; Lincke, J.; Kobalz, K.; Erhart, O.; Bergmann, J.; Lässig, D.; Lange, M.; Möllmer, J.; Gläser, R.; Staudt, R.; et al. Paddle wheel based triazolyl isophthalate MOFs: Impact of linker modification on crystal structure and gas sorption properties. *Inorg. Chem.* **2016**, *55*, 3030–3039. https://doi.org/10.1021/acs.inorgchem.5b02921.

[19] Kobalz. M, Metal-organic frameworks basierend auf 1,2,4-Triazolylisophtalaten und -benzoaten: Einfluss von Liganden- und Metallionensubstitution auf Struktur und Adsorptionseigenschaften, PhD-Thesis, Universität Leipzig, Leipzig, 24.08.2016

[20] Lange, M.; Kobalz, M.; Bergmann, J.; Lässig, D.; Lincke, J.; Möllmer, J.; Möller, A.; Hofmann, J.; Krautscheid, H.; Staudt, R.; Gläser, R. Structural flexibility of a copper-based metal–organic framework: sorption of C_4-hydrocarbons and in situ XRD. *J. Mater. Chem. A* **2014**, *2*, 8075.

[21] Coelho, A. *TOPAS*, Bruker AXS GmbH, Karlsruhe, 2014.

[22] Larson, A.C.; Von Dreele, R.B. General Structure Analysis System (GSAS); Los Alamos National Laboratory Report LAUR 86-748, 2004.

[23] Hähnel, T.; Kalies, G.; Krishna, R.; Möllmer, J.; Hofmann, J.; Kobalz, M.; Krautscheid, H. Adsorptive separation of C2/C3/C4-hydrocarbons on a flexible Cu-MOF: The influence of temperature, chain length and bonding character. *Micropor. Mesopor. Mat.* **2016**, *224*, 392–399. https://doi.org/10.1016/j.micromeso.2015.12.056.
